# A waste-to-wealth initiative exploiting the potential of *Anabaena variabilis* for designing an integrated biorefinery

**DOI:** 10.1038/s41598-022-13244-8

**Published:** 2022-06-08

**Authors:** Dipanwita Deb, Nirupama Mallick, P. B. S. Bhadoria

**Affiliations:** Agricultural and Food Engineering Department, Indian Institute of Technology Kharagpur, Kharagpur, 721302 India

**Keywords:** Biotechnology, Environmental biotechnology, Industrial microbiology, Biopolymers, Bioremediation, Analytical biochemistry, Biofuels, Bioalcohols

## Abstract

The current research work was an innovative approach providing dual advantages of waste bioremediation and an effective biorefinery. The study attempted to exploit wastewater like aqua discharge and solid wastes like poultry litter/cow dung for cyanobacterial cultivation. Aqua discharge appended with 7.5 g L^−1^ poultry litter turned out as the best combination generating 46% higher carbohydrate yield than BG-11 control. *A. variabilis* cultivation in this waste-utilized medium also revealed its excellent bioremediation ability. While 100% removal was observed for nitrite, nitrate, and orthophosphate, a respective 74% and 81% reduction was noted for ammonium and total organic carbon. Chemical and biological oxygen demands were also reduced by 90%. This work was also novel in developing a sequential design for the production of bioethanol and co-products like exopolysaccharides, sodium copper chlorophyllin, C-phycocyanin, and poly-β-hydroxybutyrate from the same cyanobacterial biomass. The developed biorefinery implementing the waste-utilized medium was one of its kind, enabling biomass valorization of 61%. Therefore, the present study would provide a leading-edge for tackling the high production costs that limit the practical viability of biorefinery projects. The recyclability of the bioremediated wastewater would not only curtail freshwater usage, the waste disposal concerns would also be mitigated to a great extent.

## Introduction

One of the daunting threats to sustainable livelihood is the increased waste accumulation and inability to curb a proper means for their disposal. Standing at the crossroads for human progressions and a clean planet, an intermediate path is indispensable to recycle the waste into ecologically viable products. Talking about circular systems, nature can be considered a seamless example. Atmospheric carbon dioxide-oxygen balance and the water cycle are such cyclic phenomena that render equilibrium among living organisms. However, at present human interaction with nature is mostly linear, where the input resources for producing goods and services are typically discarded, ending up generating a large quantity of waste that is hardly recycled.

An important part of the agricultural sector is constituted by the aquaculture industry. Globally, aquaculture is one of the rapidly expanding industries contributing to 47% of the fish supply, which in the coming decade is expected to upsurge up to 60%^1^. This globalization would generate a large volume of nutrient-laden wastewater that plays a key role in the eutrophication of water bodies. Ironically, the traditionally employed wastewater treatment plants being cost-intensive with inefficient nutrient recycling does not ensure the removal of these pollutants. With the increasing population, the demand for livestock (including poultry) has also accelerated considerably^2^. Livestock rearing generates substantial wastes from feed materials, excrements, and farm leftovers, which can be environmentally detrimental if suitable disposal methods are not chalked out. Yet, much attention has not been paid to these alarming problems. For instance, the conventional poultry waste disposal practices in agricultural lands might result in overflows and run-offs in the adjacent areas due to rigorous farming and excessive dumping^3^. There have also been reports on groundwater contamination arising from extensive usage of poultry manure in crop fields^4,5^.

In the context of waste management, anaerobic digestion has been employed for a long to treat organic matters by translating them into biogas primarily constituted of CH_4_ and CO_2_^6,7^. However, there remains a vast possibility that these greenhouse gases escape into the environment due to biogas mishandlings. Also, practical applications of the produced biogas mandate its purification, which would involve extra cost, thereby questioning its economic sustainability^8^. Therefore, in the recent past, research was underway to transform waste into more value-added and ecologically sustainable products than biogas. Several high-value intermediates, such as volatile fatty acids (VFAs), are generated during the acidogenesis phase of the anaerobic digestion as a result of organic matter breakdown and microbial fermentation^9^. Microbially derived VFAs are basically short-chain carboxylic acids (C_2_–C_4_), including acetic, lactic, propionic, butyric, and succinic acids serving as building blocks for an array of industrially significant products^6,10^. Medium-chain VFAs (C_6_–C_8_) can also be obtained by anaerobic fermentation using mixed culture of microorganisms by the process of chain elongation^11^. Thus, by restricting the methanogenesis phase, it is possible to attain increased accretion of these intermediates, prohibiting their conversion into CH_4_ and CO_2_^12^. Although animal wastes like cow/dairy manure can be a potential substrate for VFAs production by this method^13,14^, intense pretreatment and digestion steps are essential to degrade the intractable part composed of lignocellulosic substances^7,15^. Furthermore, specific process controls involving modifications of the operational parameters are required to inhibit methanogenesis and induce VFAs accumulation^6^.

Irrespective of these complexities, cyanobacterial cultivation system can be a cost-effective way to promote efficient waste remediation and an active biorefinery. Cyanobacterial growth and biomass production are dependent on the availability of nutrients, with nitrogen and phosphate sources as their primary requirement^16^. Wastewater and livestock wastes, a rich source of these nutrients, can thus be utilized for cyanobacterial cultivation. The capability of these organisms to utilize organic matter also promises to lessen the organic load from these wastes^17^. At the same time, the cyanobacterial biomass can be harnessed for the generation of biofuel and valuable co-products. The waste-utilization efficiency of cyanobacteria has been studied by some researchers, also demonstrating their nutrient removal potential. In a study by Mezzomo et al.^18^, *Spirulina platensis* demonstrated a specific growth rate of 0.4 d^−1^ when grown in swine wastewater supplemented Zarrouk medium. The same study also recorded 43.3% reduction in phosphorus and 84.3% decrease in COD (Chemical oxygen demand). In another such work, CFTRI (Central Food Technological Research Institute) medium appended with cow dung ash was used to cultivate *S. platensis*, where a biomass yield of 1.2 g L^−1^ was obtained. The percentage removal of TSS (Total suspended solids), TDS (Total dissolved solids), and COD were 8.1%, 51%, and 83.6%, respectively^19^. Likewise, *Phormidium* sp. also caused effective bioremediation of tannery effluent (obtained from leather industry), resulting in a respective 87.6%, 76.2%, 86.9%, 79.1%, and 50.7% removal of total nitrogen (TN), total phosphorus (TP), BOD (Biological oxygen demand), COD, and TDS^20^. In a more recent work^21^, *Leptolyngbya* sp. dominated microbial consortium was grown in electrochemically pretreated brewery wastewater. The said study documented 91.6%, 98.5%, and 89.4% reductions in COD, TP, and TN, respectively, with biomass produce of 0.74 g L^−1^.

Despite these handful studies, more extensive research is required to explore the direct application of livestock wastes alongside wastewater for cyanobacterial cultivation. Formulating an inexpensive growth medium combining different types of wastes is expected to annul the necessity of incorporating additional synthetic medium components. Moreover, though the refinery concept has arisen as a sustainable option, certain research gaps still exist. Generation of multiple value-added products in a single step-wise protocol, exploiting cyanobacteria cultivated using waste resources is hardly available in the literature.

Therefore, the present investigation was undertaken to formulate a waste-utilized medium using solid wastes like cow dung/poultry litter and wastewater such as aqua discharge for *Anabaena variabilis* cultivation*.* To determine the bioremediation ability of the cyanobacterium, the nutrient removal percentages were measured over time. As an effort toward converting wastes to wealth, the study also attempts to generate bioethanol and invaluable co-products from the obtained cyanobacterial biomass. The study further proposes an integrated sequential processing strategy for establishing a cyanobacterial refinery.

## Methods

### Control cultures and growth estimation

Control cultures of the nitrogen-fixing cyanobacterium *Anabaena variabilis* were grown in 100 mL BG-11 medium, modified by eliminating the incorporation of additional nitrogen source^22^. The composition of this modified BG-11 medium has been elaborated in Deb et al.^23^ The culture room for incubating the cyanobacterial culture was set at a temperature of 25 ± 2 °C, receiving 75 µmole photon m^−2^ s^−1^ PAR (14:10 h) light intensity, without carbon dioxide/air sparging. The cultures were subjected to manual shaking on a regular basis (2–3 times a day) to negate their adherence to the flask’s lower surface.

Growth measured in terms of biomass yield was estimated as dry cell weight (dcw)^24^. This involved harvesting the cyanobacterial biomass by centrifugation and allowing it to oven-dry at 60 °C taken in a vial. After the biomass attained a constant weight, its yield was calculated by deduction of the empty vial’s weight (measured initially) from the final weight of the vial.

### Estimation of total carbohydrate and its components

The amount of total carbohydrate in the test cyanobacterium was estimated by the phenol–sulfuric acid method^25^ using 5% phenol and conc. sulfuric acid. To determine the component sugars, the cyanobacterial biomass was further analyzed for reducing sugar, glycogen, starch, cellulose, and hemicellulose. The protocols for determining the total carbohydrate and its components have been expansively discussed in Deb et al.^26^.

### Cultivation of the test cyanobacterium utilizing wastes

Different wastes were chosen to test their suitability in formulating an inexpensive growth medium for cyanobacterial cultivation. These included wastewater like Aqua Discharge and solid wastes such as Poultry litter and Cow dung.

#### Characterization of aqua discharge (AD)

Aqua Discharge (AD) was collected from the Aquaculture section of the Department of Agricultural and Food Engineering, Indian Institute of Technology Kharagpur, and analyses of different parameters were carried out. These parameters viz., orthophosphate, ammonium, nitrate, nitrite, total organic carbon (TOC), biological oxygen demand (BOD), chemical oxygen demand (COD), dissolved oxygen (DO), and pH were determined following the standard methodologies provided by American Public Health Association (APHA)^27^. The content of DO was assessed with the help of a DO meter (Model no. 55-12 FT, YSI Inc., Ohio, USA), whereas a pH meter (Van London Co., USA) was used to measure the pH of the sample.

#### Bioremediation efficiency of the test cyanobacterium grown in AD

*A. variabilis* was grown in AD, and its bioremediation efficiency was analyzed over 35 days at an interval of 5 days. The bioremediation efficiency was measured as the nutrient removal capacity of the cyanobacterium along with changes in other parameters such as BOD, COD, and DO with time and expressed as percentage removal.

#### Estimation of biomass and total carbohydrate of the test cyanobacterium grown in AD

100 mL AD taken in 250 mL Erlenmeyer flasks was autoclaved and inoculated with *A. variabilis*. The cultures were maintained under the same condition as the control cultures, and time-course analysis for biomass and carbohydrate production were performed for 36 days of incubation with 3-day interval.

#### Characterization of poultry litter and cow dung

Solid wastes viz., Poultry litter (PL) and Cow dung (CD) were collected from the local cattle and poultry firms of Kharagpur, India. These were then characterized for orthophosphate, ammonium, nitrate, nitrite, and TOC following the standard APHA protocols^27^.

#### Cultivation and analysis of the test cyanobacterium under different concentrations of PL and CD

PL and CD were first ground, and different concentrations (5 g L^−1^, 10 g L^−1^, 15 g L^−1^, 20 g L^−1^) were prepared by mixing the PL/CD powder with millipore water. The suspension was vortexed well, followed by filtration using Whatman No. 44 filter paper. The PL and CD extracts of each concentration (100 mL) taken in separate flasks were then autoclaved and inoculated with *A. variabilis*. The conditions of the culture room remained same as that of the control. The biomass and total carbohydrate production of the test cyanobacterium were analyzed individually for each concentration of PL and CD every 3^rd^ day till the 36^th^ day of incubation.

### Combination of wastewater with solid wastes

The study also investigated the effects of combining different supplementations of each solid waste (PL/CD) in wastewater (AD) on the growth and carbohydrate production ability of *A. variabilis*. Based on the results, the most suitable combination was selected. The developed ‘waste-utilized (WU)’ medium was then characterized and inoculated with the test cyanobacterium, where its bioremediation efficacy was tested over the span of 35 days. Finally, the obtained biomass was analyzed for determining the carbohydrate profile to check its suitability for bioethanol production.

### Bioethanol and co-products from the test cyanobacterium grown in WU medium

Before moving ahead to the refinery concept, the potential of *A. variabilis* grown in WU medium was first assessed for the individual production of bioethanol as well as co-products such as C-phycocyanin (C-PC), poly-β-hydroxybutyrate (PHB), exopolysaccharides (EPS), and sodium copper chlorophyllin (SCC). Their extraction and estimation were carried out using the same procedure that has been followed in Deb et al.^26^ and further ascertained using various confirmatory analyses.

The production of bioethanol was confirmed by Gas chromatography and Mass spectrometry (GC–MS) (Perkin-Elmer, Shelton, CT, USA) analysis using 1-propanol as the internal standard (0.2%). The device was fitted with PE-5® phenyl methylpolysiloxane capillary column of dimension: 30 m × 0.25 mm × 0.25 µm and operated at a temperature of 40 °C initially for 1.6 min, after which it was hiked to 200 °C at a flow rate of 30 °C min^−1^. The obtained mass spectra of the sample were then interpreted by comparing with the references available in the NIST (National Institute of Standards and Technology) library.

C-PC production was confirmed spectrophotometrically (Lambda 25 UV/Vis, Perkin Elmer, Shelton, CT, USA) by detection of the peak at the absorbance maxima of C-PC when scanned within the range of 200–700 nm. While PHB obtained from *A. variabilis* was validated using GC analysis^28^, the monosaccharide composition of the extracted EPS was analyzed using HPLC^29^. The formation of SCC was confirmed using copper test and flame test as suggested by JECFA (Joint FAO/WHO Expert Committee on Food Additives)^30^. In copper test, 0.1% sodium diethyldithiocarbamate was incorporated into the SCC sample solution and checked for the formation of a brown precipitate. Flame test detected the presence of copper by observing the color transition of the flame as the test solution was burnt. To further confirm SCC production, the sample was scanned spectrophotometrically within the wavelength of 340–700 nm. The absorption spectrum of the test sample was then correlated with that of the standard SCC (Sigma Chemical Co., USA), scanned within the same range of wavelength.

### Proposing an integrated sequential processing approach for a cyanobacterial refinery

Based on the screening of different sequential extraction protocols, a cyanobacterial refinery design was proposed utilizing the WU medium. This involved step-wise production of all the cyanobacterial products (including bioethanol) from the same harvested biomass in a way to have minimal impact of extraction solvents and procedures used for one product on the yield of the other placed below in the sequence.

**Figure 1:**
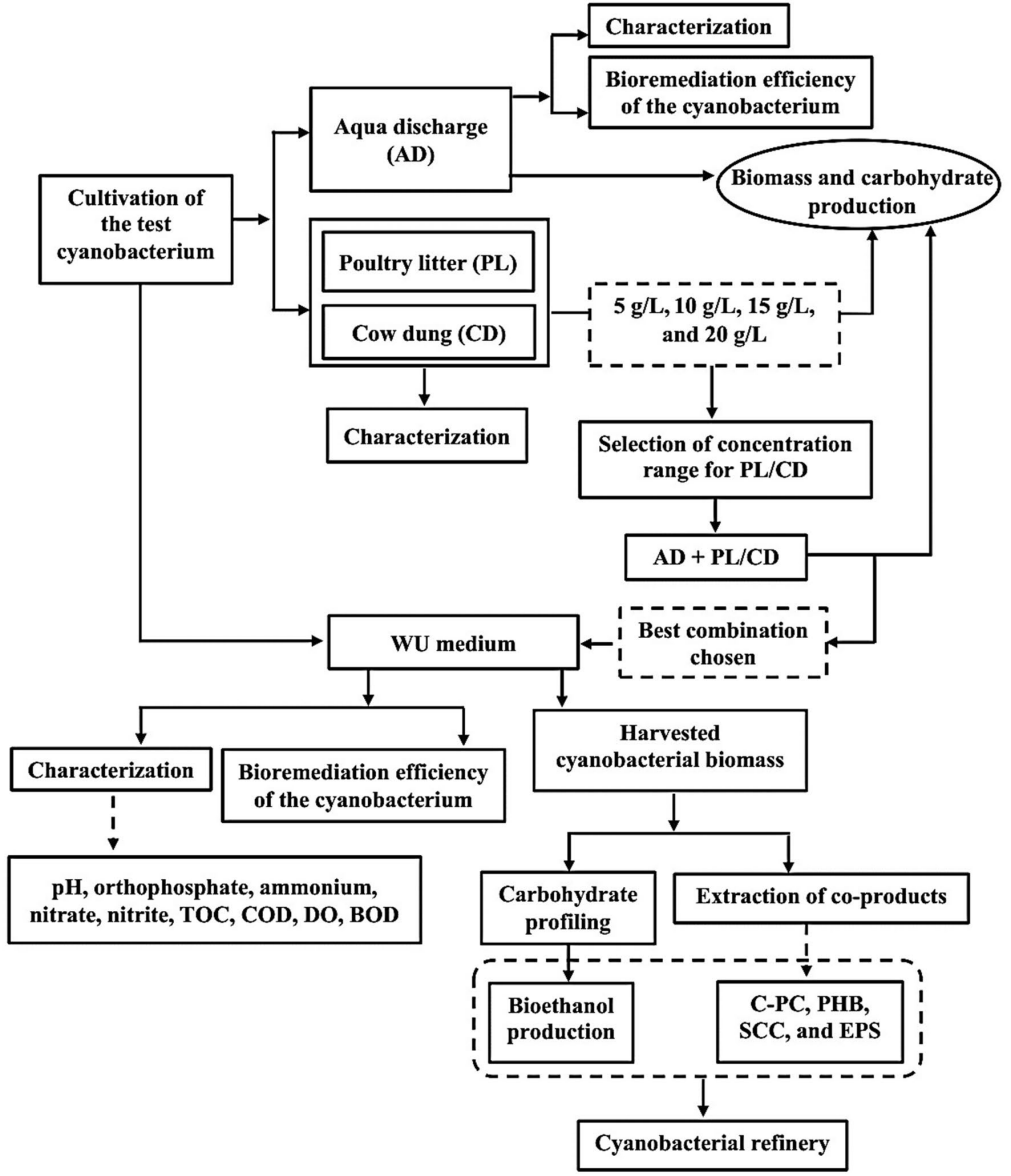
Overall experimental design for developing the ‘waste-utilized (WU)’ medium from wastewater (Aqua discharge) and solid wastes (Poultry litter/Cow dung).

The overall experimental design for developing the cost-effective WU medium using wastewater (Aqua discharge) and solid wastes (Poultry litter/Cow dung) for cultivation of *A. variabilis* and developing a cyanobacterial refinery is presented in Fig. 1.

### Statistical analysis

All the experiments were performed in triplicate and repeated three times to confirm the consistency and reproducibility of the results. Using MSTAT-C software (Plant and Soil Sciences Division, Michigan State University, USA), Duncan’s new multiple range test (DMRT) was performed for statistical evaluation of the data.

## Results

### Characterization of aqua discharge, poultry litter, and cow dung

The study began with the characterization of wastewater, and solid wastes, where all the wastes analyzed viz., aqua discharge (AD), poultry litter (PL), and cow dung (CD) were found to be enriched with total organic carbon (TOC). AD also displayed a considerable presence of nitrate with a comparatively lower amount of nitrite, orthophosphate, and ammonium. In contrast, for the solid wastes (PL and CD), ammonium and orthophosphate were much more prevalent than nitrate and nitrite. Additionally, the lower dissolved oxygen (DO) and considerable chemical oxygen demand (COD), and biological oxygen demand (BOD) values of AD indicated the presence of organic load in the samples (Tables 1 and 2).

**Table 1:** Characteristics of aqua discharge (AD).

Parameter	Concentration
Orthophosphate (mg L^−1^)	3.2 ± 0.2
Ammonium (mg L^−1^)	2.6 ± 0.1
Nitrate (mg L^−1^)	14.3 ± 1.0
Nitrite (mg L^−1^)	3.6 ± 0.3
TOC (mg L^−1^)	20.8 ± 1.3
DO (mg L^−1^)	4.3 ± 0.3
COD (mg L^−1^)	138.6 ± 4.4
BOD (mg L^−1^)	62.4 ± 2.7
pH	6.4 ± 0.4

Values were calculated as the mean (± SE) of three independent observations, i.e., n = 3.

**Table 2:** Characteristics of poultry litter (PL) and cow dung (CD).

Parameter	Content (mg g^−1^)	
	PL	CD
Orthophosphate	11.8 ± 0.62^b^	9.7 ± 0.48^a^
Ammonium	13.6 ± 0.81^b^	11.3 ± 0.62^a^
Nitrate	1.2 ± 0.05^b^	0.84 ± 0.02^a^
Nitrite	0.21 ± 0.01^b^	0.15 ± 0.01^a^
Total Organic Carbon (TOC)	14.0 ± 0.78^b^	10.3 ± 0.56^a^

Values were calculated as the mean (± SE) of three independent observations, i.e., n = 3.

Significant differences among the values (P < 0.05, DMRT) within a row are designated using different alphabetic annotations.

### Biomass and total carbohydrate estimation of *A. variabilis* cultivated in AD medium compared to control BG-11 medium

**Figure 2:**
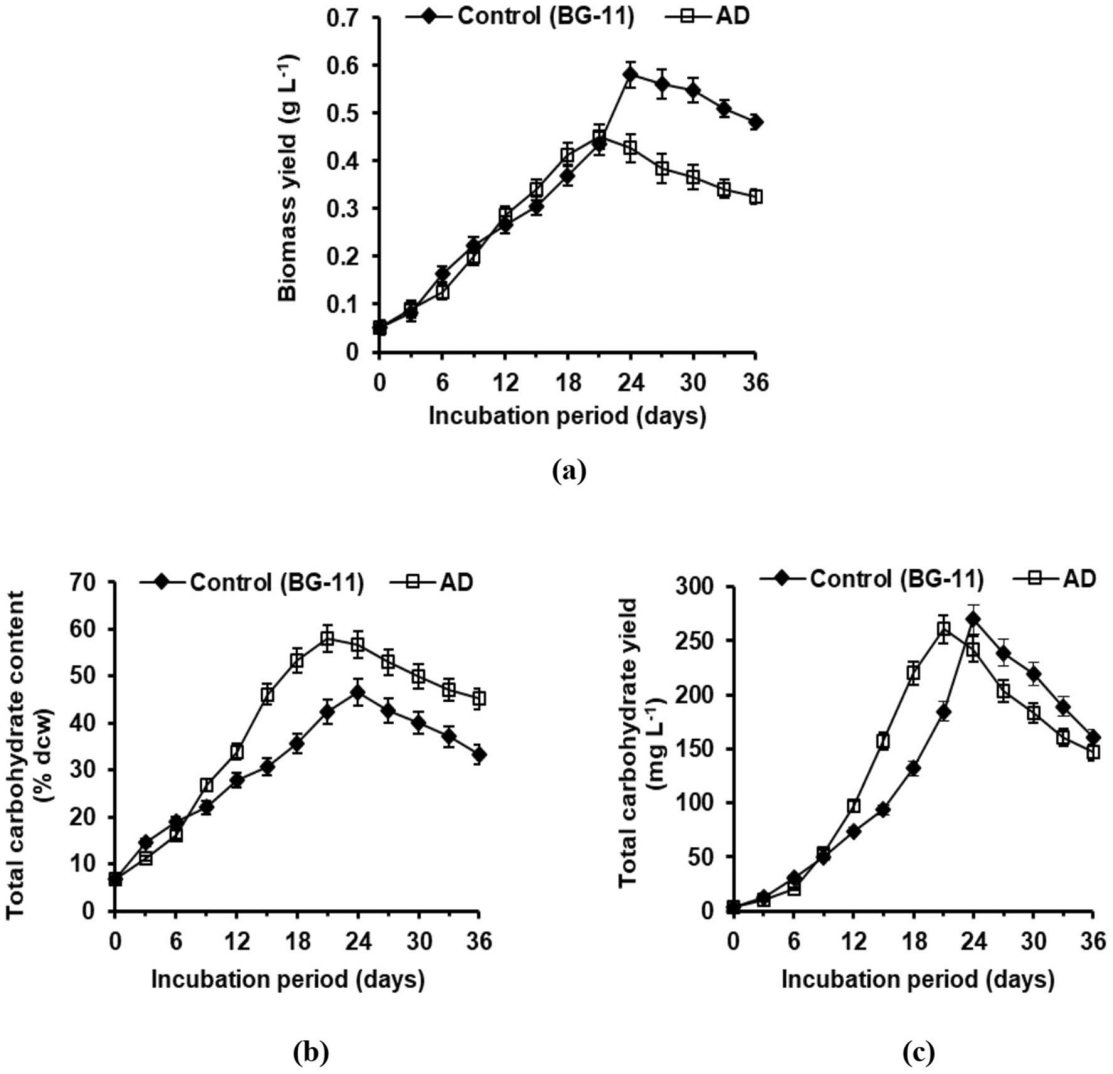
Time-course analysis of (**a**) biomass yield (g L^−1^), (**b**) total carbohydrate content (% dcw), and (**c**) total carbohydrate yield (mg L^−1^) of *A. variabilis* cultivated in AD medium compared to control BG-11 medium.

Time-course studies were conducted to compare the biomass and total carbohydrate production of *A. variabilis* in control (BG-11 medium) and AD medium (Fig. 2). In this regard, the maximum biomass yield obtained using AD medium (0.45 g L^−1^) was fairly lower than that of the control (0.58 g L^−1^) (Fig. 2a). However, the total carbohydrate content displayed a ~ 20% higher value in the AD medium (58.0% dcw) compared to control (46.5% dcw) (Fig. 2b). This increased content obtained using the AD medium compensated for the reduced biomass yield, thus, not compromising with the volumetric production (mg L^−1^) of total carbohydrate. As a result, the total carbohydrate yield was comparable in both the cultivation media with respective values of 269.7 mg L^−1^ and 261.0 mg L^−1^ in control and AD medium (Fig. 2c). In contrast to control, where the maximum value for biomass and total carbohydrate was recorded on the 24th day, the incubation period was reduced to 21st day for cultures grown in the AD medium.

### Biomass and total carbohydrate estimation of *A. variabilis* cultivated in different concentrations of PL and CD

**Figure 3:**
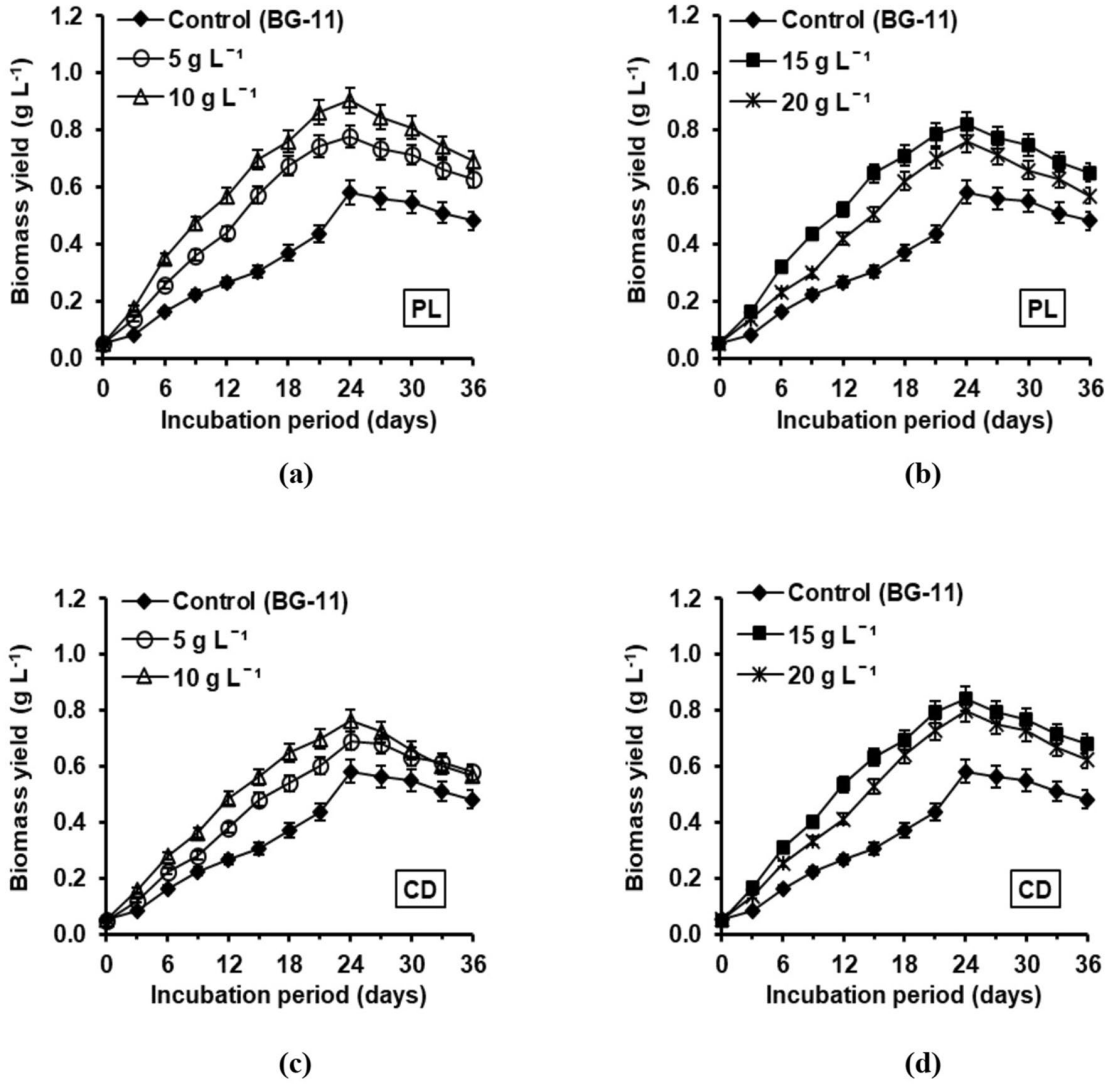
Time-course analysis of biomass yield (g L^−1^) of *A. variabilis* cultivated in different concentrations of PL: (**a**) 5 g L^−1^, 10 g L^−1^ (**b**) 15 g L^−1^, 20 g L^−1^ and CD: (**c**) 5 g L^−1^, 10 g L^−1^ (**d**) 15 g L^−1^, 20 g L^−1^ compared to control BG-11 medium.

Comparative analysis for biomass yield of *A. variabilis* cultivated in different concentrations of PL and CD are depicted in Fig. 3a–d, respectively. In order to have a better representation, two concentrations were considered at a time, each for PL and CD, and compared to the BG-11 control. An increasing trend was observed in the biomass production by augmenting the PL level up to 10 g L^−1^, whereas with CD extract, the maximum value was obtained at 15 g L^−1^ concentration, above which a declining pattern was observed. However, PL was found to stimulate the growth of *A. variabilis* more effectively than CD. The biomass yield attained its paramount value (0.90 g L^−1^) in the PL concentration of 10 g L^−1^ compared to 0.58 g L^−1^ in control (BG-11 medium), representing a 35.5% rise (Fig. 3a). On the other hand, the biomass yield reached a maximum of 0.84 g L^−1^ using the CD concentration of 15 g L^−1^, demonstrating a 30.9% higher value than the control (Fig. 3d). For all the studied concentrations, *A. variabilis* displayed the peak value on day 24 of its incubation.

Similarly, PL concentration of 10 g L^−1^ also resulted in the highest total carbohydrate content of 57.4% dcw with respect to 46.4% dcw obtained in the control BG-11 medium. Consequently, its yield was boosted to 516.6 mg L^−1^, representing a 47.9% higher value than the control (269.1 mg L^−1^) (Table 3). In contrast, for CD, the maximum total carbohydrate content of 52.3% dcw was recorded using 15 g L^−1^ concentration. At this concentration, the total carbohydrate yield reached 439.3 mg L^−1^, showing a 38.7% rise compared to control (Table 3).

**Table 3:** Maximum total carbohydrate production of *A. variabilis* cultivated in different concentrations of PL and CD, compared to control BG-11 medium.

PL/CD concentration	Total carbohydrate production using PL		Total carbohydrate production using CD	
	Content (% dcw)	Yield (mg L^−1^)	Content (% dcw)	Yield (mg L^−1^)
Control (BG-11)	46.4 ± 2.1^b^	269.1 ± 7.4^a^	46.4 ± 2.1^b^	269.1 ± 7.4^a^
5 g L^−1^	48.1 ± 2.3^b^	375.2 ± 7.9^b^	47.8 ± 2.2^b^	329.8 ± 7.7^c^
10 g L^−1^	57.4 ± 2.6^c^	516.6 ± 8.4^d^	49.6 ± 2.3^b^	377.0 ± 7.9^d^
15 g L^−1^	51.6 ± 2.4^b^	423.1 ± 8.1^c^	52.3 ± 2.4^b^	439.3 ± 8.1^e^
20 g L^−1^	36.8 ± 1.7^a^	279.7 ± 7.5^a^	38.1 ± 1.7^a^	304.8 ± 7.5^b^

Values were calculated as the mean (± SE) of three independent observations, i.e., n = 3.

Significant differences among the values (P < 0.05, DMRT) within a column are designated using different alphabetic annotations.

### Studies with combination of wastewater with solid wastes

#### Biomass and total carbohydrate estimation of *A. variabilis* cultivated in wastewater supplemented with different concentrations of PL and CD

The individual studies with solid wastes conducted so far involved cultivating the test cyanobacterium in PL and CD extract prepared with millipore water. To further economize the process, subsequent analyses were made in a medium prepared by supplementing these solid wastes in wastewater (AD). AD supplemented with 7.5 g L^−1^ PL was found to be the most suitable combination resulting in the highest biomass yield of 0.85 g L^−1^. Augmenting the level of PL in the AD medium to 10 g L^−1^ marked its de-escalation to 0.77 g L^−1^ (Table 4). For CD supplemented conditions, the combination of AD with 10 g L^−1^ CD was best amongst all, resulting in biomass produce of 0.78 g L^−1^ (Table 4). However, PL supplementation once again had a more dominating effect on *A. variabilis*, generating higher biomass yield at a comparatively lower concentration (7.5 g L^−1^). The biomass yield using the said combination (AD + 7.5 g L^−1^ PL) represented a 31.8% rise compared to the BG-11 control, which was also 47.1% higher than the un-supplemented AD medium.

**Table 4:** Maximum biomass and total carbohydrate production of *A. variabilis* in AD supplemented with different concentrations of PL and CD, compared to control (BG-11) and un-supplemented AD.

PL concentration	Biomass yield (g L^−1^)	Total carbohydrate		CD concentration	Biomass yield (g L^−1^)	Total carbohydrate	
		Content (% dcw)	Yield (mg L^−1^)			Content (% dcw)	Yield (mg L^−1^)
Control (BG-11)	0.58 ± 0.01^b^	46.4 ± 2.1^a^	269.1 ± 7.4^a^	Control (BG-11)	0.58 ± 0.01^b^	46.4 ± 2.1^a^	269.1 ± 7.4^a^
AD	0.45 ± 0.01^a^	57.9 ± 2.6^b^	260.1 ± 7.3^a^	AD	0.45 ± 0.01^a^	57.9 ± 2.6^a^	260.1 ± 7.3^a^
AD + 5 g L^−1^	0.64 ± 0.02^c^	47.5 ± 2.2^a^	304.0 ± 7.5^b^	AD + 5 g L^−1^	0.59 ± 0.01^b^	46.7 ± 2.1^a^	275.5 ± 7.5^a^
AD + 7.5 g L^−1^	0.85 ± 0.03^e^	58.4 ± 2.6^b^	496.4 ± 8.3^d^	AD + 10 g L^−1^	0.78 ± 0.03^c^	53.4 ± 2.4^a^	416.5 ± 8.1^c^
AD + 10 g L^−1^	0.77 ± 0.03^d^	54.8 ± 2.4^b^	422.0 ± 8.1^c^	AD + 15 g L^−1^	0.73 ± 0.03^c^	49.5 ± 2.2^a^	361.4 ± 7.8^b^

Values were calculated as the mean (± SE) of three independent observations, i.e., n = 3.

Significant differences among the values (P < 0.05, DMRT) within a column are designated using different alphabetic annotations.

The carbohydrate production of the test cyanobacterium was also found to be maximum in AD supplemented with 7.5 g L^−1^ PL. The total carbohydrate content of 58.4% dcw and yield of 496.4 mg L^−1^ obtained using this combination was again highest in comparison to all the other PL supplemented conditions (Table 4). These values were also higher than the CD supplemented AD medium, where the maximum total carbohydrate content (53.4% dcw) and yield (416.5 mg L^−1^) were obtained at a concentration of 10 g L^−1^ (Table 4). Hence, using AD in combination with 7.5 g L^−1^ PL, a respective rise of 20.5% and 45.8% in total carbohydrate content and yield was recorded with respect to the control BG-11 medium. Although, compared to the un-supplemented AD medium, the cellular carbohydrate content did not show much variation, the rise in its volumetric yield was by 47.6%. Therefore, for the economical production of large volume bioethanol, the combination of AD with 7.5 g L^−1^ PL was chosen for further study. This medium was termed as ‘APL.’

#### Characterization of APL medium

The obtained APL medium was also characterized for the presence of orthophosphate, ammonium, nitrate, and nitrite, TOC, DO, COD, BOD and pH which are depicted in Table 5.

**Table 5:** Characteristics of APL medium.

Parameter	Concentration
Orthophosphate (mg L^−1^)	67.8 ± 2.8
Ammonium (mg L^−1^)	74.2 ± 2.9
Nitrate (mg L^−1^)	20.6 ± 1.3
Nitrite (mg L^−1^)	4.5 ± 0.4
TOC (mg L^−1^)	91.3 ± 3.6
DO (mg L^−1^)	4.1 ± 0.3
COD (mg L^−1^)	161.3 ± 5.1
BOD (mg L^−1^)	73.2 ± 2.9
pH	6.0 ± 0.4

Values were calculated as the mean (± SE) of three independent observations, i.e., n = 3.

#### Bioremediation efficiency of *A. variabilis* grown in APL medium

Inoculation of the APL medium with *A. variabilis* revealed 100% removal of orthophosphate within 30 days; and nitrate and nitrite within 20 and 15 days of incubation, respectively. While the concentration of ammonium was reduced to 19.2 mg L^−1^ from an initial value of 74.2 mg L^−1^, TOC content showed a drop from 91.3 mg L^−1^ to 17.8 mg L^−1^ in 35 days of incubation. In other words, cultivation of the test cyanobacterium in the APL medium brought about a respective 74.1% and 80.5% depletion in its ammonium and TOC concentration. COD and BOD were also reduced to 16.6 mg L^−1^ and 7.3 mg L^−1^ at the end of 35 days compared to 161.3 mg L^−1^ and 73.2 mg L^−1^, respectively, on day zero. Concurrently, an increase in the DO up to 6.8 mg L^−1^ was observed after the completion of 35 days of incubation (from 4.1 mg L^−1^ noted initially). The maximum pH of 7.3 was recorded on the 25th day of incubation (Table 6).

**Table 6:** Concentration changes in APL medium after inoculation with *A. variabilis*.

Parameter (mg L^−1^)	Incubation period (days)							
	0	5	10	15	20	25	30	35
Orthophosphate (mg L^−1^)	67.8 ± 2.8^f^	48.1 ± 2.2^e^	36.2 ± 1.9^d^	21.3 ± 1.5^c^	9.2 ± 0.8^b^	3.82 ± 0.3^a^	ND	ND
Ammonium (mg L^−1^)	74.2 ± 2.9^h^	66.8 ± 2.7^g^	58.6 ± 2.4^f^	45.8 ± 2.1^e^	39.5 ± 1.9^d^	33.5 ± 1.8^c^	27.3 ± 1.8^b^	19.2 ± 1.7^a^
Nitrate (mg L^−1^)	20.6 ± 1.3^d^	14.7 ± 1.1^c^	7.2 ± 0.6^b^	2.1 ± 0.2^a^	ND	ND	ND	ND
Nitrite (mg L^−1^)	4.5 ± 0.4^c^	2.0 ± 0.2^b^	1.2 ± 0.1^a^	ND	ND	ND	ND	ND
TOC (mg L^−1^)	91.3 ± 3.6^h^	70.9 ± 2.9^g^	63.1 ± 2.5^f^	52.9 ± 2.3^e^	44.1 ± 2.1^d^	35.0 ± 1.8^c^	28.1 ± 1.7^b^	17.8 ± 1.6^a^
DO (mg L^−1^)	4.1 ± 0.3^a^	5.2 ± 0.3^b^	5.8 ± 0.3^b^	6.4 ± 0.5^b^	6.6 ± 0.5^b^	6.7 ± 0.6^b^	6.7 ± 0.5^b^	6.8 ± 0.6^b^
COD (mg L^−1^)	161.3 ± 5.1^h^	119.5 ± 4.5^g^	101.4 ± 3.7^f^	72.7 ± 2.9^e^	59.6 ± 2.5^d^	38.9 ± 2.0^c^	23.1 ± 1.6^b^	16.6 ± 1.5^a^
BOD (mg L^−1^)	73.2 ± 2.9^h^	56.6 ± 2.5^g^	47.2 ± 2.2^f^	33.7 ± 1.9^e^	25.9 ± 1.7^d^	16.0 ± 1.5^c^	10.1 ± 0.8^b^	7.3 ± 0.6^a^
pH	6.0 ± 0.4^a^	6.2 ± 0.4^a^	6.4 ± 0.4^a^	6.7 ± 0.5^a^	7.1 ± 0.5^a^	7.3 ± 0.6^a^	7.2 ± 0.5^a^	7.1 ± 0.5^a^

Values were calculated as the mean (± SE) of three independent observations, i.e., n = 3.

ND = Not detected.

Significant differences among the values (P < 0.05, DMRT) within a row are designated using different alphabetic annotations.

### Individual production of bioethanol and co-products from *A. variabilis* grown in APL medium

As already observed in Table 4, AD + 7.5 g L^−1^ PL induced the maximum carbohydrate accumulation in *A. variabilis*. The carbohydrate obtained from the test cyanobacterium grown in APL medium was also predominated by higher yields of fermentable components such as reducing sugar and glycogen (Supplementary Figure S1). Consequently, an elevated volumetric production of bioethanol was obtained using the APL medium. Compared to 118.4 mg L^−1^ in BG-11 control, the bioethanol yield obtained from APL-grown *A. variabilis* showed a rise to 219.9 mg L^−1^, signifying a 46.2% higher value. Nevertheless, for both the conditions (control and APL), the bioethanol conversion % was found to range between 44 and 44.3% (Table 7).

**Table 7:** Bioethanol production in control (BG-11) and APL medium along with the conversion efficiencies.

Cultivation medium	Total carbohydrate yield (mg L^−1^)	Bioethanol yield (mg L^−1^)	Bioethanol conversion %
Control (BG-11)	269.1 ± 7.4^a^	118.4 ± 5.5^a^	44.0 ± 1.7^a^
APL	496.4 ± 8.7^b^	219.9 ± 6.6^b^	44.3 ± 1.8^a^

Values were calculated as the mean (± SE) of three independent observations, i.e., n = 3.

Significant differences among the values (P < 0.05, DMRT) within a column are designated using different alphabetic annotations.

APL medium also served effectively for significant accumulation of all the co-products (Table 8). Using this medium, C-PC content was increased to 5.4% dcw from 3.0% dcw in BG-11 control. On the other hand, PHB and SCC contents were respectively enhanced to 8.3% and 0.07% dcw in the APL medium compared to 6.1% and 0.04% dcw in control. A marginal increase was also witnessed in the EPS content from 39.2% (control) to 43.6% dcw (APL medium). This upsurge in the cellular concentration of the co-products alongside the augmented biomass yield of *A. variabilis* in the APL medium also heightened their volumetric production. A ~ 61–62% increase was noted for SCC and C-PC yield, recording respective values of 0.59 mg L^−1^ and 45.9 mg L^−1^ in the APL medium compared to 0.23 mg L^−1^ and 17.4 mg L^−1^ in BG-11 control. Likewise, the yield of PHB and EPS were also elevated to 70.6 mg L^−1^ and 370.6 mg L^−1^ from 35.4 mg L^−1^ and 227.4 mg L^−1^ in control, indicating a respective ~ 50% and ~ 39% rise (Table 8).

**Table 8:** Co-products obtained individually from *A. variabilis* grown in the BG-11 control and APL medium, expressed as content (% dcw) and yield (mg L^−1^).

Co-products	Content (% dcw)		Yield (mg L^−1^)	
	Culture conditions		Culture conditions	
	Control	APL	Control	APL
C-PC	3.0 ± 0.4^a^	5.4 ± 0.7^b^	17.4 ± 1.2^a^	45.9 ± 2.5^b^
PHB	6.1 ± 0.8^a^	8.3 ± 1.2^b^	35.4 ± 2.0^a^	70.6 ± 4.1^b^
SCC	0.04 ± 0.001^a^	0.07 ± 0.004^b^	0.23 ± 0.01^a^	0.59 ± 0.05^b^
EPS	39.2 ± 2.1^a^	43.6 ± 2.2^b^	227.4 ± 7.1^a^	370.6 ± 8.9^b^

Values were calculated as the mean (± SE) of three independent observations, i.e., n = 3.

Significant differences among the values (P < 0.05, DMRT) within a row are designated using different alphabetic annotations. Each row was analyzed separately for content and yield.

**Figure 4:**
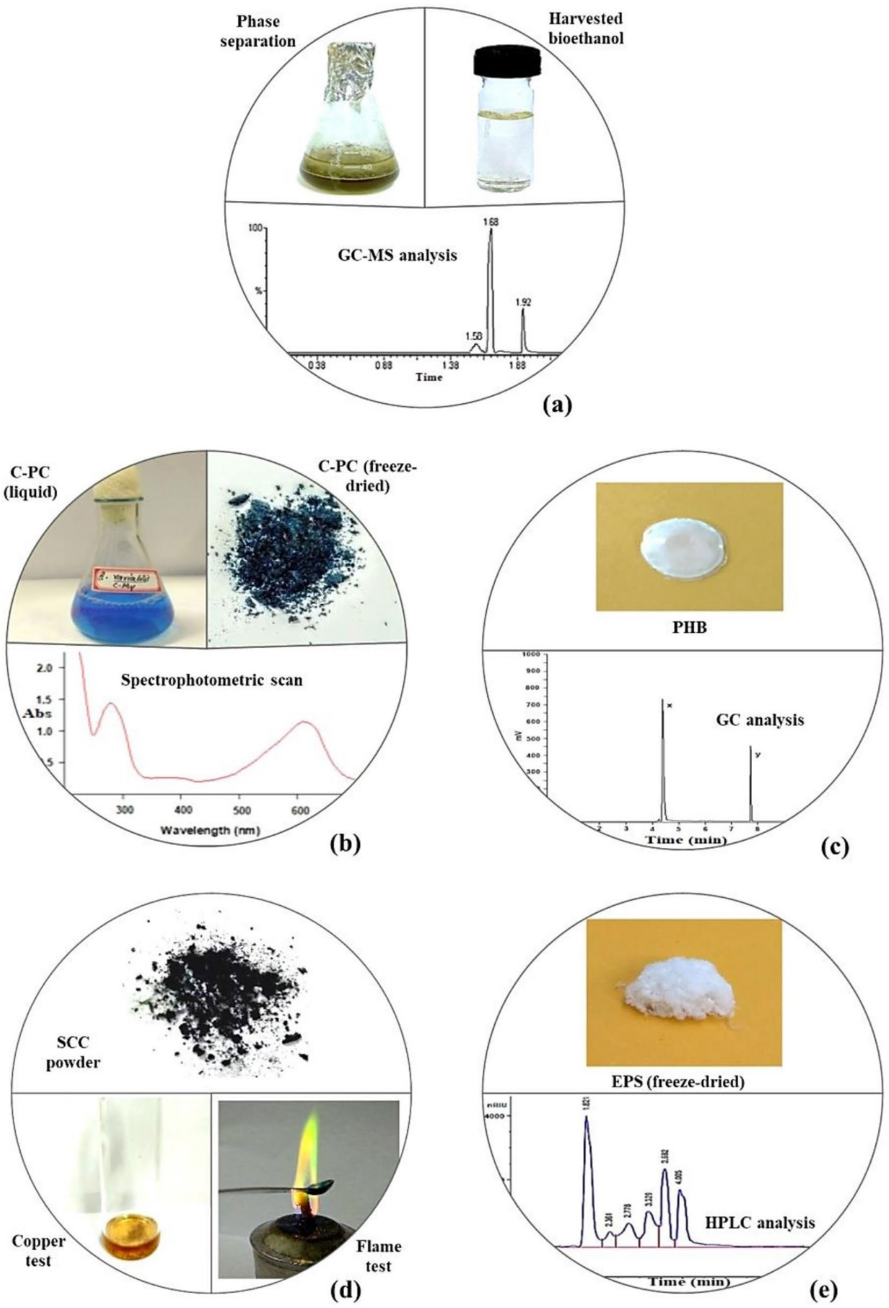
Production and confirmatory analyses of (**a**) Bioethanol, (**b**) C-PC, (**c**) PHB, (**d**) SCC, and (**e**) EPS from *A. variabilis*.

Bioethanol and the co-products harnessed from *A. variabilis* and their validation using different confirmatory analyses have been summarized in Fig. 4, the comprehensive illustration of which has been provided in Supplementary Figures S2–S7.

Bioethanol was obtained after fermentation of the pretreated hydrolysate and harvested by phase separation (Fig. 4a) using Tri-n-butyl phosphate (TBP). The bioethanol production was confirmed using GC–MS analysis, where its presence was detected at the retention time of 1.68 min (Fig. 4a and Supplementary Figure S2), referring to the NIST mass spectral library.

C-phycocyanin (C-PC) was extracted as water-soluble blue color pigment and stored as freeze-dried powder (Fig. 4b). The confirmatory detection of C-PC was based on the spectrophotometric determination of the distinct peak at 620 nm, the absorption maxima of C-PC (Fig. 4b and Supplementary Figure S3).

Poly-β-hydroxybutyrate (PHB) was extracted as white polymer, and its accumulation in *A. variabilis* was detected using GC analysis (Fig. 4c). The polymer sample obtained from the test cyanobacterium conformed to the standard PHB exhibiting the specific peak at the retention time of 4.5 min, hence, confirming PHB synthesis by *A. variabilis* (Supplementary Figure S4).

Chlorophyll extracted from *A. variabilis* was converted into its water-soluble derivative- sodium copper chlorophyllin (SCC), which appeared as blue-black powder (Fig. 4d). The identification of SCC was performed using two confirmatory tests (copper test and flame test) to validate the presence of copper in it. In copper test, the formation of a brown-colored complex of copper diethyldithiocarbamate after addition of sodium diethyldithiocarbamate confirmed the presence of copper in the produced SCC sample. In flame test, the SSC sample solution burnt with a green-colored flame, hence, reconfirming the presence of copper in it (Fig. 4d). Additionally, the absorption spectrum of the produced SCC sample was also found to be similar to the standard SCC, showing peaks at the wavelengths of 405 nm and 635 nm (Supplementary Figure S5). This analogy in absorbance behavior again validated the SCC formation in the present study.

Exopolysaccharides (EPS) extracted from the test cyanobacterium when freeze-dried appeared as white cottony fiber (Fig. 4e). HPLC analysis revealed the obtained EPS to be constituted of pentoses like ribose and xylose; and hexoses, such as mannose, glucose, and galactose (Supplementary Figure S6), demonstrating the characteristic feature of cyanobacterial EPS^29^. The identification of these constituent sugars was performed by comparing their elution time with that of the standards (Supplementary Figure S7a–e).

### Cyanobacterial refinery

After having established the suitability of APL medium in stimulating the individual yields of bioethanol and the co-products, it was applied to sequentially extract all the cyanobacterial products from the same harvested biomass. However, the major hindrance faced while executing a step-wise production is the intervention of solvents and extraction processes employed for a particular product on the yield of the others that are to be extracted subsequently. Therefore, in this study, six different sequences were analyzed to screen out the best amongst them that would ensure minimum yield losses (Table 9).

**Table 9:**
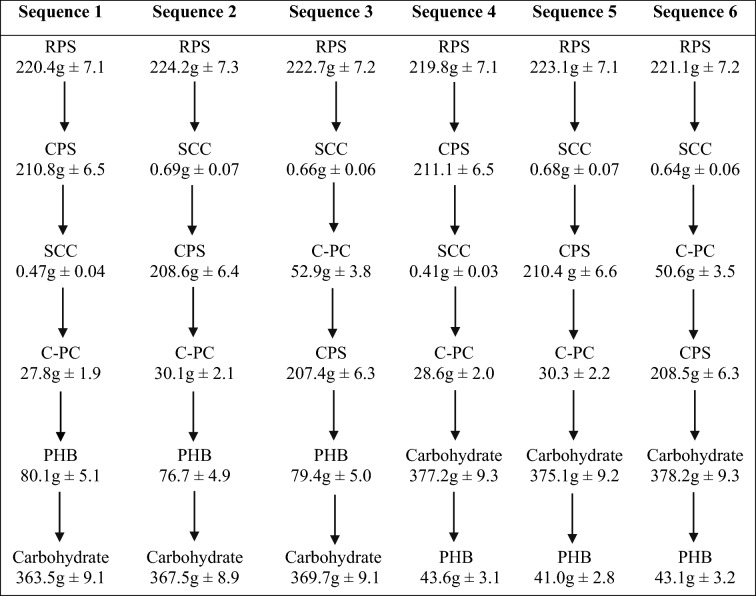
Variation in the yield of different products from *A. variabilis* (equivalent to 1 kg dry biomass) grown in APL medium when placed in different sequential extraction positions.

Values were calculated as the mean (± SE) of three independent observations, i.e., n = 3.

RPS = released polysaccharides; CPS = capsular polysaccharides; Total EPS = RPS + CPS, where EPS refers to exopolysaccharides.

**Figure 5:**
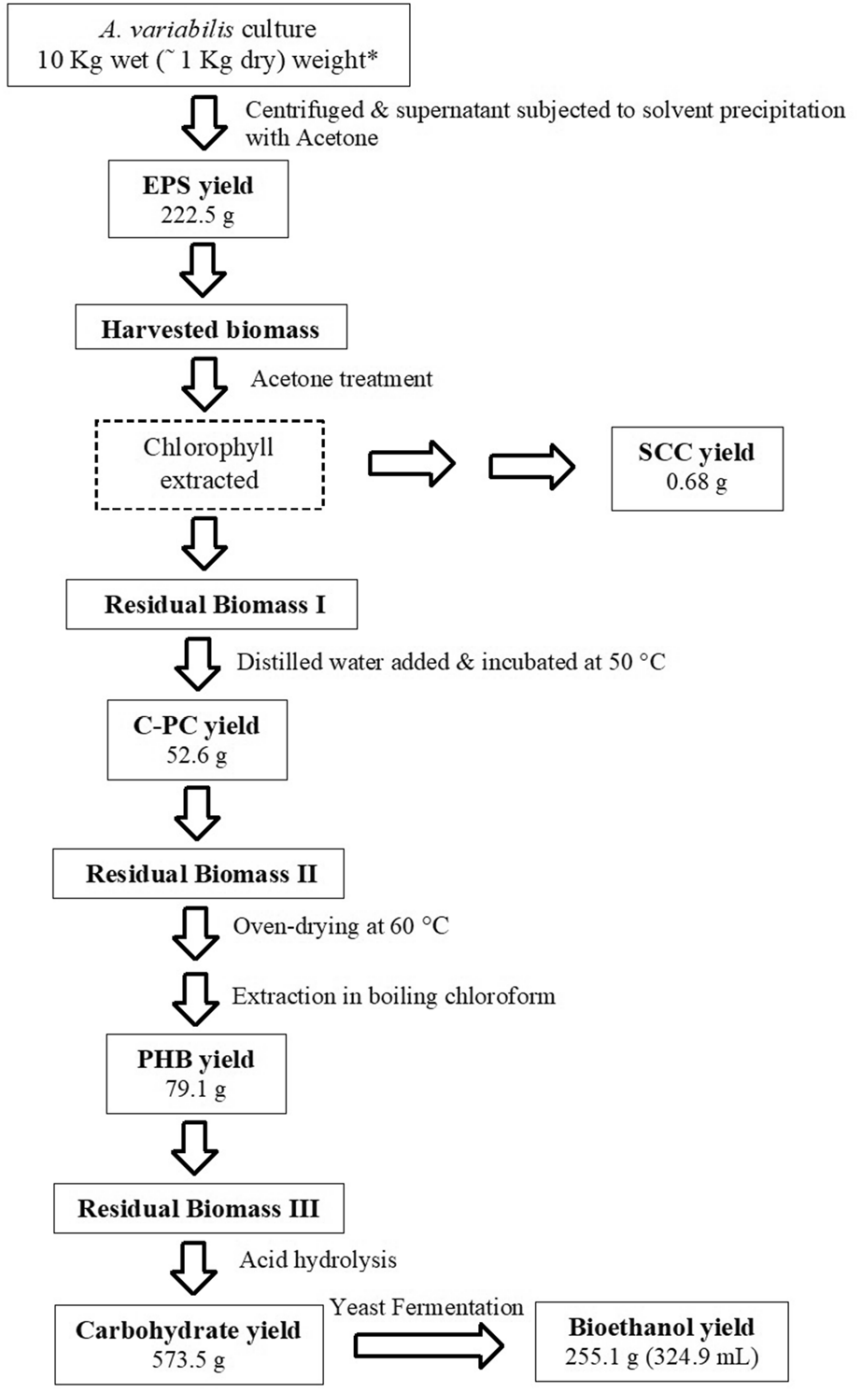
Detailed process of sequential extraction of different products from *A. variabilis* under cyanobacterial refinery approach. *1 kg dry biomass corresponds to ≈1176 L culture volume.

Here, the amount of the harnessed products was calculated considering 1 kg dry (≈10 kg wet) biomass of *A. variabilis* grown in the APL medium. Hence, it becomes mandatory first to mention the yields of each product obtained individually from 1 kg of dry biomass without implementing the sequential extraction strategy. The individual EPS yield was 436.0 g, constituted by 224.5 g RPS and 211.5 g CPS, while the yields for SCC, C-PC, PHB, and carbohydrate were 0.69 g, 54.0 g, 83.1 g, and 584.0 g, respectively. Comparison of all the studied sequences (Table 9) revealed sequence 3 to result in minimum yield loss of all the co-products, comparable to their individual yields. Using this sequence, EPS yield of 430.1 g (222.7 g RPS + 207.4 g CPS), SCC yield of 0.66 g, C-PC yield of 52.9 g, and PHB yield of 79.4 g was obtained. However, compared to individual yield, carbohydrate was compromised in all the sequences, with sequence 3 generating a yield of 369.7 g. This was possibly owing to the prior extraction of CPS (capsular polysaccharides), which accounts for a significant portion of the cell’s total carbohydrate. As a consequence, the anticipated bioethanol yield was likely to be hampered, which is the primary product of the study. Therefore, the carbohydrate and bioethanol production from *A. variabilis* was also checked under the selected sequence 3 by eliminating the CPS extraction step. Removing this step resulted in notably higher bioethanol production (324.9 mL) comparable to the individual yield obtained from 1 kg dry biomass (329.5 mL). With respect to the sequence integrating CPS extraction, the bioethanol yield using the modified protocol (without CPS) also indicated a 35% rise (Supplementary Table S1). As a whole, using this designed refinery approach, ~ 61% of the cyanobacterial biomass could be effectively utilized for the production of bioethanol and various industrially vital products. The detailed design for the cyanobacterial refinery using the selected sequence has been schematically illustrated in Fig. 5.

### Techno-economic assessment

One of the critical factors that confine the practical implication of a biorefinery is its economic feasibility under large-scale. Hence an idea regarding the techno-economic aspect is vital to apprehend the success of the developed strategy when upgraded to a large-scale scenario. Considering the direct costs involved in a biomass-based biorefinery, huge expenses are incurred in the growth medium of the test species that serve as the raw material for biomass production^31^. Nonetheless, in our current approach, the cyanobacterial species exploits waste-utilized APL medium for its growth, thereby completely nullifying the cost of the cultivation medium. The efficient nutrient removal ability of *A. variabilis* further alleviates the expenditure on additional waste remediation infrastructures^32^. Moreover, since the wastes (for formulating APL medium) were collected from the close vicinity of the experimental site, the transportation charges can also be considered negligible.

However, the harvesting process of the cyanobacterial biomass is a major cost-incurring step that can hinder large-scale biomass production. Hence, proper choice of harvesting methodology is crucial while aiming for biomass harvest under a scaled-up system. Among the different techniques, pH-induced flocculation using NaOH has been reported as the most economical and eco-friendly harvesting method for large-scale set-ups^33,34^. Assuming a scaled-up cultivation system of 10,000 L employing the APL medium, the harvest cost is estimated to be 0.765 $. This assessment relies on the published investigation of Koley et al.^33^, demonstrating the requirement of ~ 62 g NaOH/kg of harvested biomass alongside power consumption of 0.2 kW h/kg for culture agitation under field-level analysis.

Implementing the cyanobacterial refinery would also take into account the investments on the solvents and chemicals for extraction of the products. Following the sequential biorefinery protocol designed in this study, the cost of these extractants per Kg biomass reaches 7420.8 US$ (Table 10). It is worth mentioning here that the expenditure on pre-treatment, fermentation, and separation of bioethanol involving the use of sulfuric acid, yeast (*Saccharomyces cerevisiae*), YPD (yeast extract peptone dextrose) medium, and tributyl phosphate (TBP)^26^ would also contribute to the overall cost (Table 10).

**Table 10:** Cost estimation of chemicals and solvents required for harnessing the cyanobacterial products using the sequential biorefinery design, utilizing APL medium.

Extraction solvents and chemicals	Cost incurred (US$/kg)^a^
Acetone	6997.2
Sodium hydroxide	58.8
Copper sulfate	4.3
Hydrochloric acid	61.5
Chloroform	182.2
Sulfuric acid	1.8
Yeast for fermentation	0.376
YPD medium components	0.52
TBP	114.1
Total	7420.8

^a^Cost was evaluated considering per kg of dry biomass obtained using the APL medium, based on the price of bulk chemicals from http://www.alibaba.com.

Hence, from the above table, the estimated cost of extractants required for the scaled-up biorefinery (10,000 L culture volume) can be evaluated as 63,102 US$. Additionally, the refinery protocol also involves oven drying of the cyanobacterial biomass (Fig. 5). With electricity requirement of ~ 6.32 kWh for obtaining 1 kg dry biomass^26,35^, the cost for drying the biomass harvested from 10,000 L culture approximates to 7.52 US$.

Despite these outlays, the large-scale execution of the proposed biorefinery looks plausible in view of the high commercial value of the harnessed cyanobacterial products. The projected earnings from commercializing all the cyanobacterial products (EPS, SCC, C-PC, PHB, and bioethanol) obtained under a scaled-up scenario of the present approach corresponds to ~ 7.2 million US$. This estimation has been carried out considering the market price of each product^23,26^. However, the total production cost would also include the cost of the cultivation set-up. Closed systems like a tubular photobioreactor (PBR) have been reported to account for up to 83% of the capital cost^36^. Besides, such mass-cultivation operations have to meet with general overhead charges related to cleaning, maintenance, and administration of the plant^31^. Though open-pond systems are known to procure lesser investments, PBRs ensure higher productivity accompanied by low downstream processing charges^23,37^. Hence, keeping aside some days for cleaning and maintenance of the system, running multiple cycles of cultivation per year would definitely increase the profit margin. This would, in turn, recompense the cost of initial investments on large-scale projects, helping to overcome the real-time challenges faced while establishing a successful biorefinery.

### Environmental sustainability assessment

Environmental sustainability is another significant area to be addressed while assessing the aptness of a developed strategy. One way to conduct this assessment is through the evaluation of impact indicators such as cumulative primary energy (CPE) and global warming potential (GWP)^38^. Using the LCA (Life Cycle Analysis) methodology with these impact indicators, all the inputs and outputs of a system are taken into account. The output constitutes both quantitative and qualitative products generated, alongside the waste discharges in the environment, including emissions. On the other hand, the inputs encompass all the primary raw materials involved in each step of the production process. Fossil fuel for electricity generation, feedstocks for fertilizers production, energy requirement for extraction, processing, and transportation of the primary growth nutrients of the dedicated crop, etc., all fall under this category^39^. Therefore, determining the energy efficiency of a system is imperative for evaluating its ecological impact. In this direction, EROI (Energy Return on Investment) is a potential measure to check the energy balance of a particular process/product/system, which can be calculated using the following equation^40^:

$$EROI = \frac{Product\;energy\,output}{{Primary\;energy\;input\,(CED)}}$$

where ‘Product energy output’ indicates the energetic biomass fraction from which the final energy output (microalgal/cyanobacterial product) has been generated, and ‘CED’ stands for cumulative energy demand.

The above elucidation has been made in the context of third-generation feedstocks (microalgae/cyanobacteria), where a significant contribution to CED arises from the demand for nitrogen and phosphorus, the principal nutrients for their growth^41^. The value of the EROI should be > 1 to reflect a net positive energy balance^41^. This can be achieved by adopting strategies to lower the CED, which would, in turn, increase the EROI. Hence utilization of the nutrient-laden APL medium formulated out of wastes is expected to boost the overall EROI value by abating the nutrient supply in the cultivation phase. The carbon richness of the APL medium (Table 5) would also lessen the energy burden by rendering additional CO_2_ sparging inessential. Use of the flocculation method for biomass harvest can further reduce the energy consumption involved in the centrifugation step, upsurging the net EROI^42^.

There always exists a relationship between the energy inputs and greenhouse gas (GHG) emissions. The Global warming potential (GWP) (expressed as CO_2_ equivalents) serves as an important tool for environmental sustainability measurement of a particular process/product. Unfortunately, most present-day biofuel production using conventional crops contributes minimally to GHG mitigation. Cultivation of the terrestrial crops demands clearance of grasslands and forests, which results in the loss of ongoing carbon dioxide capture^43^. The soil organic matter is also broken down, accelerating the release of carbon from the soil^44^. Besides, N_2_O emissions from the field applications of fertilizers and pesticides are the foremost contributor to GHG, with GWP > CO_2_ by 298 times^45^. Contrarily, cyanobacteria/algae-derived biofuels have been specified to reduce GHG releases, particularly lowering the emissions from changes in the land-use practices^43^. The use of fertilizers and pesticides is also prevented^46^, reducing the N_2_O release. However, the emissions from fossil-fuel-driven energy production cannot be disregarded, as electricity requirement remains indispensable for various operational stages of the production process. The GWP per kWh will depend upon the electricity mix of a particular region^47^. Nonetheless, from the LCA perspective, an integrated production system with quantitative coproduct generation is known to lessen the extent of ecological impact^48^. This again points toward the large-scale applicability of the APL mediated biorefinery design, as it induced a remarkable rise in the yield of all the cyanobacterial products. Moreover, using cyanobacterial cultivation as a means of nutrient removal avoids the energy investment on separate wastewater treatment procedures, also withdrawing the release of the related emissions.

Cyanobacterial cultivation can suffer a major setback, specifically in areas where freshwater is limited. Hence, utilization of wastewater medium can also be a boon in lessening the freshwater inputs for the cultivation process. Taking ‘Water Footprints (WF)’ as an indicator of water-use extent, application of wastewater instead of freshwater would reduce the blue WF. Furthermore, the biologically treated wastewater can be recycled and reused. This would also help lower the grey WF by abating the chances of freshwater pollution^23^. A comprehensive take on the sustainability analysis of a multiproduct cyanobacterial biorefinery study would still mandate the complete LCA of each product. This would involve the evaluation of all the input and output parameters for every step right from cultivation, harvest, downstream processing, product generation, use, and disposal. A detailed analysis in this direction will be an essential domain to follow up in the offing.

**Figure 6:**
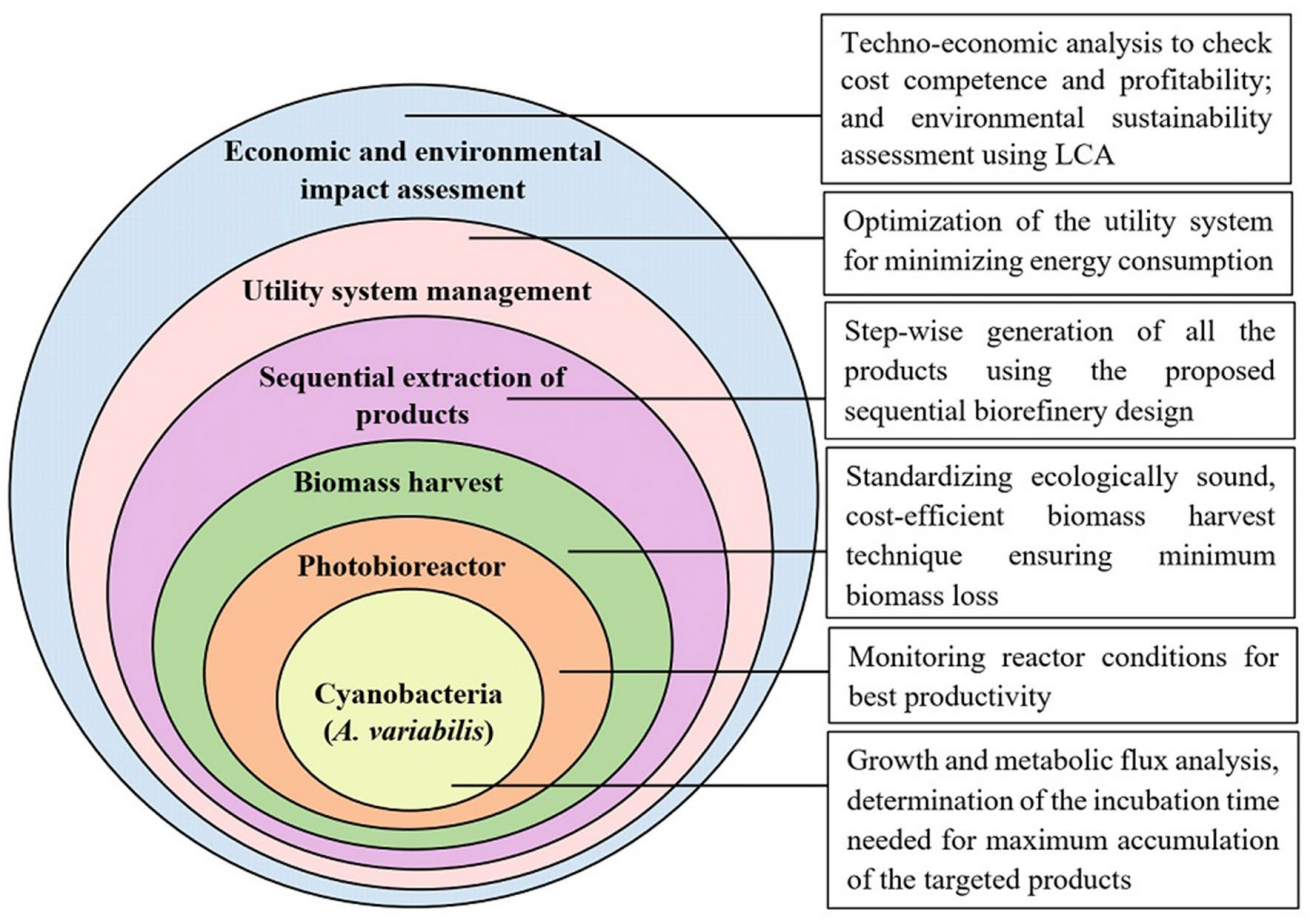
Onion model for the cyanobacterial refinery with each level assigned to specific purpose.

Nonetheless, herein we try to portray the scope of the present biorefinery design by an ‘onion model’ consisting of distinct layers (Fig. 6). This holistic interpretation gives a clear idea regarding the different levels involved in the process implementation, indicating the specific purpose of each level. It is to be noted that the onion model depicted here has been constructed in view of the futuristic prospects of the present biorefinery design when scaled up to a photobioreactor (PBR) system. The positive scopes of cultivating *A. variabilis* in a PBR set-up have been discussed in our previous paper^23^.

## Discussion

The present study explores the waste-utilization ability of *A. variabilis* exploiting aqua discharge, poultry litter, and cow dung. Aqua discharge (AD) recording a higher level of TOC can be linked to organic matter accumulation in aquaculture systems such as an aquarium in response to fish feeding^49^. Though a substantial part of the organic matter is composed of organic carbon, nitrogenous components also constitute an important portion. Therefore, when these organic matters are broken down, they release ammonia, nitrite, and nitrate as by-products. The presence of orthophosphate in the wastewater sample might also be ascribed to the breakdown of fish wastes, including uneaten fish food, feces, remains of dead fishes, etc.^50,51^. Along with TOC, the significant levels of BOD and COD with lower DO also indicate the presence of organic load in the studied sample. Similarly, solid waste like poultry litter (PL), basically a combination of residual poultry feed, poultry manure, and wastes generated in the poultry farm, makes it a potential nutrient-rich organic source^52^. On the other hand, the nutrient concentrations of animal excreta like cow dung (CD) are determined mainly by the composition of the ruminant’s diet and their intake level^53^.

When cultivated in AD medium, the growth of *A. variabilis* was compromised to some extent in comparison to BG-11 control (Fig. 2a). This can be attributed to the lower phosphate availability in AD compared to the standard BG-11 medium. Phosphorus is amongst the essential macronutrients for the growth of cyanobacteria/microalgae^54^, the limitation of which can have a degenerative effect on their biomass yield. However, application of AD medium induced ~ 20% higher total carbohydrate content (% dcw) in the test cyanobacterium (Fig. 2b). The relation between phosphate deficiency and increased cellular carbohydrate content can be interpreted from the action of ADP-glucose pyrophosphorylase (ADPGlc PPase), the key enzyme regulating the synthesis of storage carbohydrates. In the absence of Pi, ADPGlc PPase is activated by 3-phosphoglycerate, diverting the metabolic route towards the synthesis of non-phosphorylated polyglucans^55^. The enhanced carbohydrate accumulation can also be partly accredited to the carbonaceous matters present in the wastewater, which was indicated by the TOC, BOD, and COD values. These observations are in agreement with the study of Peng et al.^56^, where increased carbohydrate content of *Chlorella vulgaris* was reported under the mixotrophic growth condition using organic matters in the domestic wastewater. The elevated cellular carbohydrate content using AD medium, thus, compensated for the reduced biomass yield of the test cyanobacterium. As a result, the overall carbohydrate yield (mg L^−1^) remained un-hampered and comparable to the BG-11 control (Fig. 2c). Nonetheless, since adequate bioethanol production requires enhanced volumetric carbohydrate production, solid wastes such as PL and CD were considered for cyanobacterial cultivation in the next phase of the study.

A notable rise in the growth and carbohydrate production of *A*. *variabilis* was recorded when cultivated separately in PL and CD extracts (Fig. 3 and Table 3). The maximum biomass and total carbohydrate accumulation were obtained using 10 g L^−1^ of PL, whereas, for CD, 15 g L^−1^ concentration proved to be the most suitable. The enhanced biomass production by increasing the PL and CD levels can be linked to the higher abundance of nitrogen and phosphorus to be utilized by the test cyanobacterium. The presence of carbon compounds further provides a mixotrophic condition, stimulating higher biomass production^57^. Although compared to BG-11 control, the respective rise in cellular carbohydrate content was by ~ 19% and ~ 11%, the concurrent increase in biomass yield amplified the overall volumetric carbohydrate production (mg L^−1^) by 47.9% and 38.7%. However, these wastes being rich in carbonaceous matter, an increase in their concentrations beyond a certain level had a negative impact on the growth and carbohydrate synthesis of *A*. *variabilis*. This might be due to substrate inhibition caused by an excessive supply of exogenous carbon^58^.

The investigation next proceeded towards supplementing the solid wastes in wastewater (AD), thus cutting off freshwater intakes for the cyanobacterial cultivation. In this respect, AD + 7.5 g L^−1^ PL was the most suitable combination that resulted in maximum biomass and carbohydrate production (Table 4). Using this condition, a respective rise of 31.8% and 20.5% for biomass and total carbohydrate content was achieved compared to the BG-11 control. This enhanced the volumetric yield (mg L^−1^) of total carbohydrate by 45.8% than the control BG-11 medium. Contrarily, using CD supplemented conditions, the rise in the carbohydrate volume was only up to 35.3% (compared to control) observed for AD + 10 g L^−1^ CD. Nonetheless, using the combination of solid wastes with wastewater, the maximum values were attained at a comparatively lower concentration than using PL and CD alone. There exists an ideal level for most of the nutrients, exceeding which might have inhibitory effects on cell growth^59^. Combining wastewater with solid wastes would avail higher nutrients to the cyanobacterial species leading to the attainment of maximum biomass and carbohydrate accumulation at a comparatively lower level of supplementation.

AD + 7.5 g L^−1^ PL chosen as the best combination (termed as ‘APL’ medium), when inoculated with *A. variabilis*, displayed efficient bioremediation (Table 6). The test cyanobacterium demonstrated a 100% removal of orthophosphate, nitrate, and nitrite within 30, 20, and 15 days of incubation, respectively. COD and BOD were also reduced by 90% within 35 days incubation period. These observations derive support from the findings of Cañizares-Villanueva et al.^60^, where *Phormidium* sp. cultivated in pig waste effluent showed 100% removal of phosphate, 87% removal of nitrate, and 91% reduction in COD. The 74.1% reduction in the ammonium concentration can also be co-related with the study of Abe et al.^61^, reporting *Trentepohlia aurea* to remove 80% of ammonium from wastewater after 40 days. The depletion in the TOC content of APL by 80.5% again ascertained the test cyanobacterium’s ability to utilize TOC as carbon source. The fall in BOD and COD also reflects the consumption of organic matter by *A*. *variabilis*. The rise in the DO and pH of the APL medium was a possible consequence of cyanobacterial photosynthesis involving the uptake of CO_2_ and release of oxygen during the experimental period^62^.

The developed APL medium was also effective in supporting cyanobacterial refinery involving the sequential generation of different co-products (EPS, SCC, C-PC, and PHB) alongside bioethanol. However, rather than directly stepping into the refinery concept, individual production of each product was first assessed (Tables 7 and 8). Carbohydrate being the fermentable substrate for bioethanol production, its increased accumulation in the test species elevated the bioethanol production by 46.2%, compared to the BG-11 control. The higher levels of fermentable components like reducing sugar and glycogen in the carbohydrate obtained using APL medium (Supplementary Figure S1) explains the ease of yeast’s action during the fermentation process inducing amplified bioethanol yield^26^. The considerable increase in C-PC and SCC can be related to the improved accumulation of photosynthetic pigments in *A. variabilis* utilizing the nutrient-enriched condition of the APL medium. These observations are at par with the studies of Vadiveloo et al.^63^ and Nwoba et al.^64^, where enhanced accumulation of phycocyanin and chlorophyll were documented for cyanobacterial/microalgal cultures grown in nutrient-laden wastewater medium. Finally, the stimulation in PHB and EPS production in the APL medium can be explained based on the availability of carbon in the waste-utilized medium^65,66^.

The APL medium also proved prolific in laying down the integrated biorefinery involving sequential generation of all the cyanobacterial products. Among the six sequential designs analyzed, the order of extraction in sequence 3 was the most favorable (Table 7). In contrast to other sequences where the yield of C-PC (sequences 1, 2, 4, and 5), SCC (sequences 1 and 4), and PHB (sequences 4, 5, and 6) were compromised, sequence 3 showed analogous production to their individual yields. Herein, ‘individual yield’ refers to the amount of each product obtained independently from the entire cyanobacterial biomass. The reduced C-PC and SCC production in the respective sequences (mentioned above) can be accredited to their positioning after the CPS extraction step involving formaldehyde treatment of the biomass. Formaldehyde, known to cause immense molecular cross-linking, makes the process of protein extraction from the cell challenging^67^*.* Besides, cell shrinkage is a general phenomenon witnessed in aldehyde treated samples^68^ that might interfere with the diffusion of the pigments outside the cell during the extraction. The depleted PHB yield in sequences 4, 5, and 6 might be due to the detrimental impact of acid hydrolysis on the biomass, possibly causing leakage of its contents*.*

Although sequence 3 demonstrated a negligible loss in the yield of the co-products, carbohydrate production was affected like all the other sequences. Since RPS was extracted from the supernatant during the initial harvesting process of the wet biomass, its contribution to the total carbohydrate of the biomass would be inconsiderable. Therefore, the depleted carbohydrate yield in these sequences is a possible consequence of prior deduction of EPS in the form of CPS, bound to the cell surface. EPS reported to be chiefly constituted of carbohydrates^69^ provides a strong justification for this fact. Nevertheless, bioethanol being our key interest, any loss in the carbohydrate yield cannot be afforded as this would hinder the quantity of bioethanol produced. Hence, the investigation was then carried a step ahead by eliminating the CPS extraction step from the chosen sequence 3 (Supplementary Table S1). As expected, this modified protocol (without CPS) fetched a higher amount of carbohydrate for *S. cerevisiae* fermentation. Accordingly, the bioethanol production rose by 35% than the CPS included protocol, with its yield comparable to the individual yield. Thus, using this sequential approach, we were able to effectively translate ~ 61% of the cyanobacterial biomass into high-value merchandises (Fig. 5).

The present paper also gives an overview of the economic and environmental sustainability assessment of the developed biorefinery strategy (“Techno-economic assessment” and “Environmental sustainability assessment ”sections). Using techno-economic and LCA approaches, a positive outlook could be deduced for scaled-up application of the current strategy in the future. Though the detailed LCA of every step of the production process remains an anticipated objective of the follow-up investigation, the scope of the cyanobacterial refinery has been defined using an ‘onion model.’ The concept of the multi-layered onion diagram incorporated in this study finds support from the text of Martinez-Hernandez et al.^70^, where a biorefinery system has been illustrated from the standpoint of process engineering.

## Conclusions

On the concluding note, the APL medium formulated completely out of wastes would not only nullify the dependency on the freshwater resources but would also be an economical solution to tackle the large-scale cultivation cost. The simultaneous bioremediation of wastes in the medium would further increase its sustainability in terms of recycling and reuse of the de-polluted water. The integrated biorefinery design established in this research work is one of the major highlights of the study. The devised sequential extraction protocol exploiting the waste-utilized APL medium would undoubtedly be a cost-effective breakthrough in achieving maximum biomass utilization with minimal yield losses. Moreover, the broader range of the products generated from *A. variabilis* under the biorefinery approach endorses its relative affluence. The remunerations earned by commercializing them would ease the economic burden, especially on scaling up the process in the future.

To further improve productivity, the future prospects of the study would include switching the cultivation method from batch to semi-continuous or continuous mode. Although subjected to technical and economic constraints, simultaneous focus on obtaining a higher quantity of value-added co-products by manipulating the steady-state tactics as well as innovating hybrid designs can be promising for commercial applications of such cultivation modes. Therefore, considering these points, it would be interesting to note the performance of the test species under different cultivation approaches (viz., continuous and semi-continuous) utilizing the waste-utilized APL medium. Employing a PBR system offering the scope of operating diverse cultivation modes would certainly be a crucial futuristic endeavor to look upon. Also, a comprehensive Life Cycle Analysis (LCA) of the scaled-up biorefinery is to be performed, paving the way to an ecologically sustainable system.

## Supplementary Information

Supplementary Information.

## Data Availability

All the data supporting the present investigation are provided within this paper as well as in the Supplementary information file submitted along with this manuscript.
