# Supplementary material for: A waste-to-wealth initiative exploiting the potential of Anabaena variabilis for designing an integrated biorefinery
Source: Sci Rep. 2022 Jun 8;12:9478. doi: 10.1038/s41598-022-13244-8 (PMC9177571; doi:10.1038/s41598-022-13244-8)
Supplement: Supplementary file 1 — Supplementary Information. [file 41598_2022_13244_MOESM1_ESM.pdf]

A waste-to-wealth initiative exploiting the potential of *Anabaena variabilis* for designing an integrated biorefinery

Dipanwita Deb, Nirupama Mallick*, P. B. S. Bhadoria

Agricultural and Food Engineering Department, Indian Institute of Technology Kharagpur, Kharagpur- 721302, India

*Corresponding author.

Agricultural and Food Engineering Department,

Indian Institute of Technology Kharagpur,

Kharagpur- 721302, India; Tel.: +91 3222 283166

E-mail address: nm@agfe.iitkgp.ac.in (N. Mallick)

Supplementary Table

Supplementary Table S1. Comparison of carbohydrate and bioethanol production under the selected sequence (sequence 3) with/without CPS extraction

**With CPS**	**Without CPS**
RPS<br>222.7g ± 7.2	RPS<br>222.5 g ± 7.1
↓	↓
SCC<br>0.66g ± 0.06	SCC<br>0.68g ± 0.07
↓	↓
C-PC<br>52.9g ± 3.8	C-PC<br>52.6g ± 3.8
↓	↓
CPS<br>207.4g ± 6.3	PHB<br>79.1g ± 4.9
↓	↓
PHB<br>78.6g ± 4.9	**Carbohydrate**<br>**573.5g ± 11.2**
↓	⇣
**Carbohydrate**<br>**369.7g ± 9.1**	**Bioethanol**<br>**255.1g ± 7.4 (324.9 mL)**
⇣	
**Bioethanol**<br>**165.7g ± 5.8 (211.1 mL)**	

*Individual bioethanol yield: 258.7g (329.5 mL)

Supplementary Figures

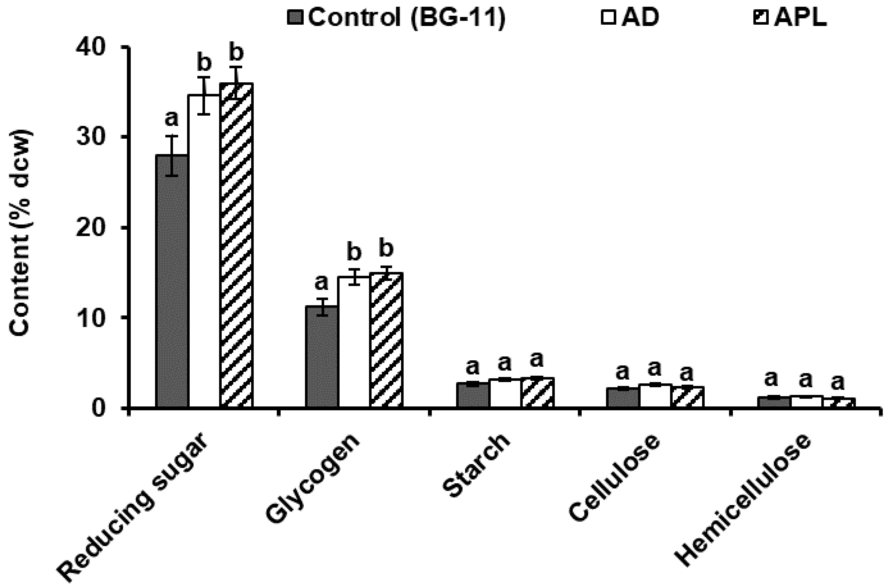

(a)

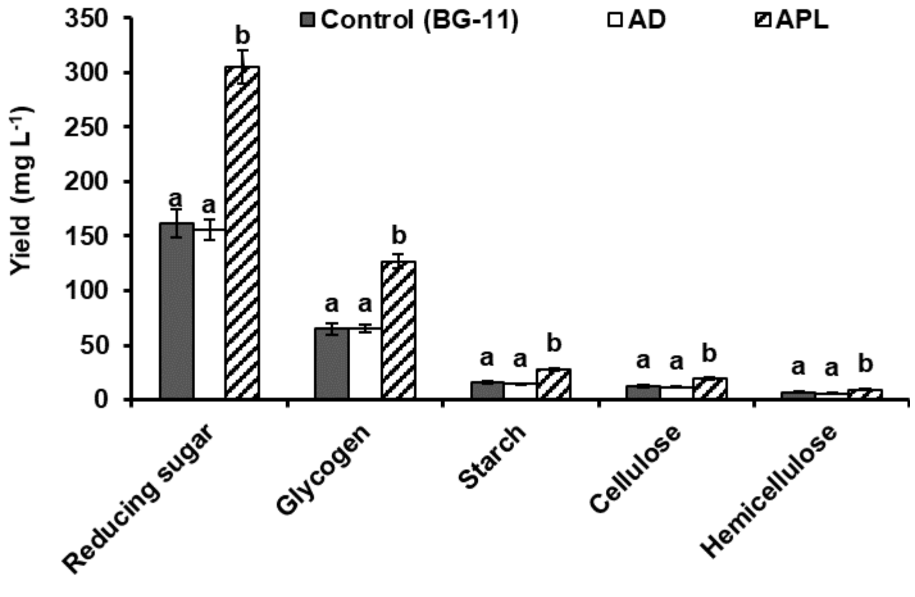

(b)

Supplementary Figure S1. Maximum **(a)** content (% dcw) and **(b)** yield (mg L^{-1}) of carbohydrate components obtained from *A. variabilis* grown in control (BG-11), AD, and APL medium. Error bars correspond to the standard deviation of three different observations (n = 3). Values differing significantly (P < 0.05, DMRT) are indicated by different alphabets over the bars, separately for each carbohydrate component.

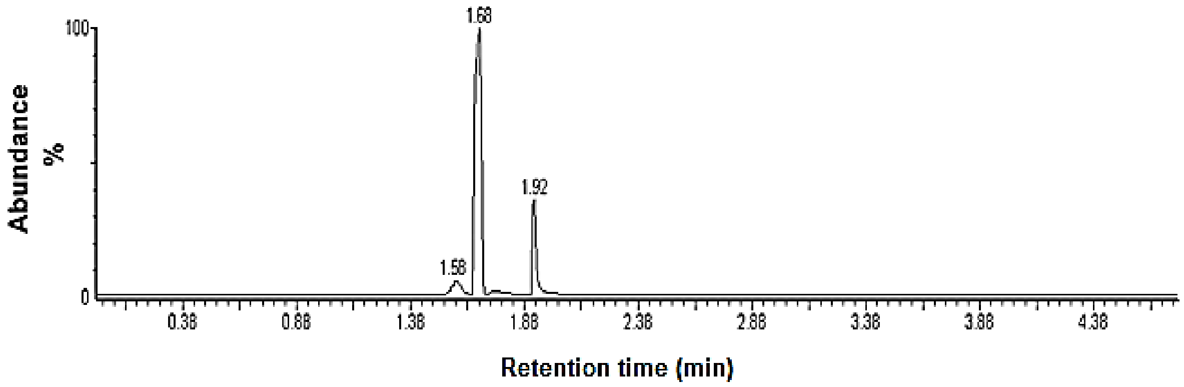

Supplementary Figure S2. GC-MS chromatogram of bioethanol sample obtained from *A. variabilis* indicating ethanol at the retention time of 1.68 min, with water and 1-propanol (internal standard) at 1.58 and 1.92 min, respectively.

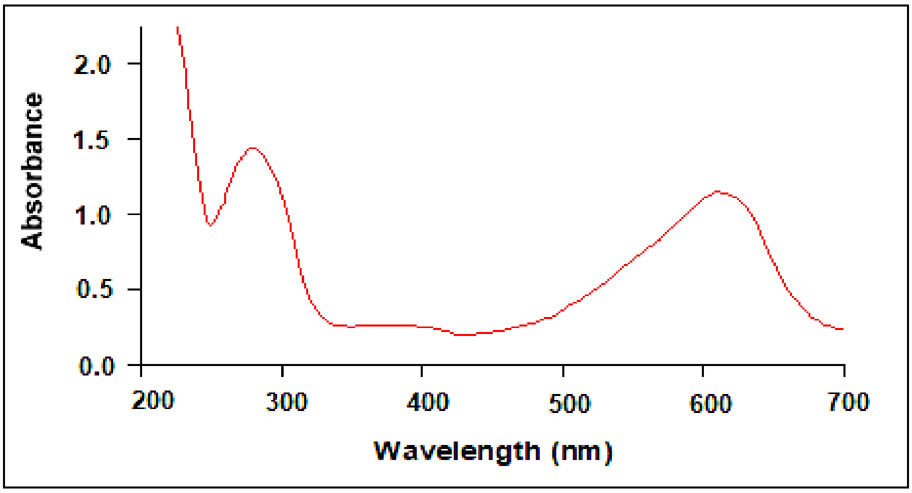

Supplementary Figure S3. Absorption spectrum of C-PC obtained using UV-Vis spectrophotometer.

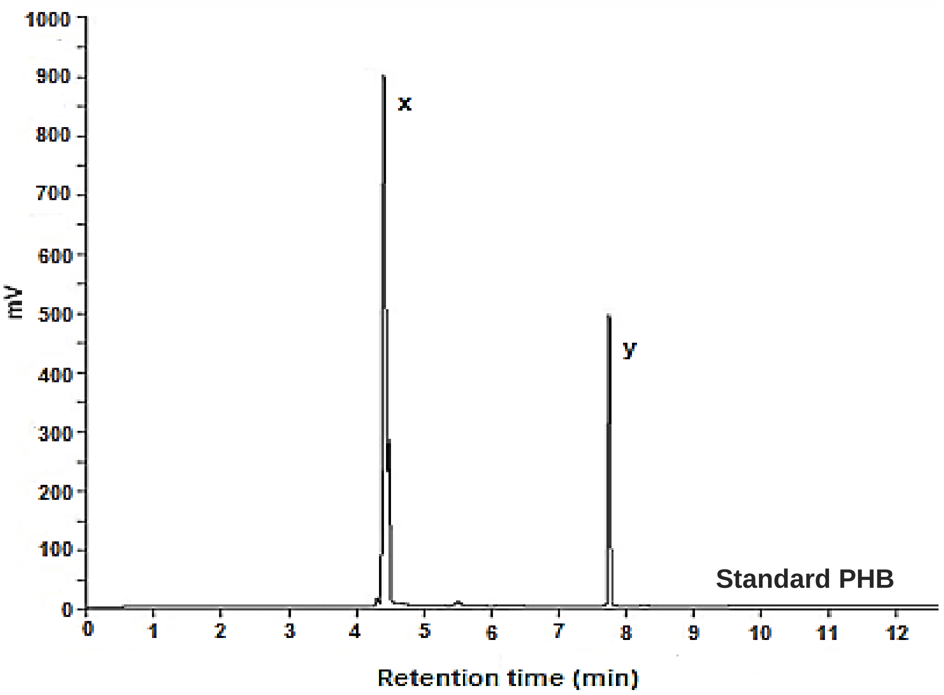

(a)

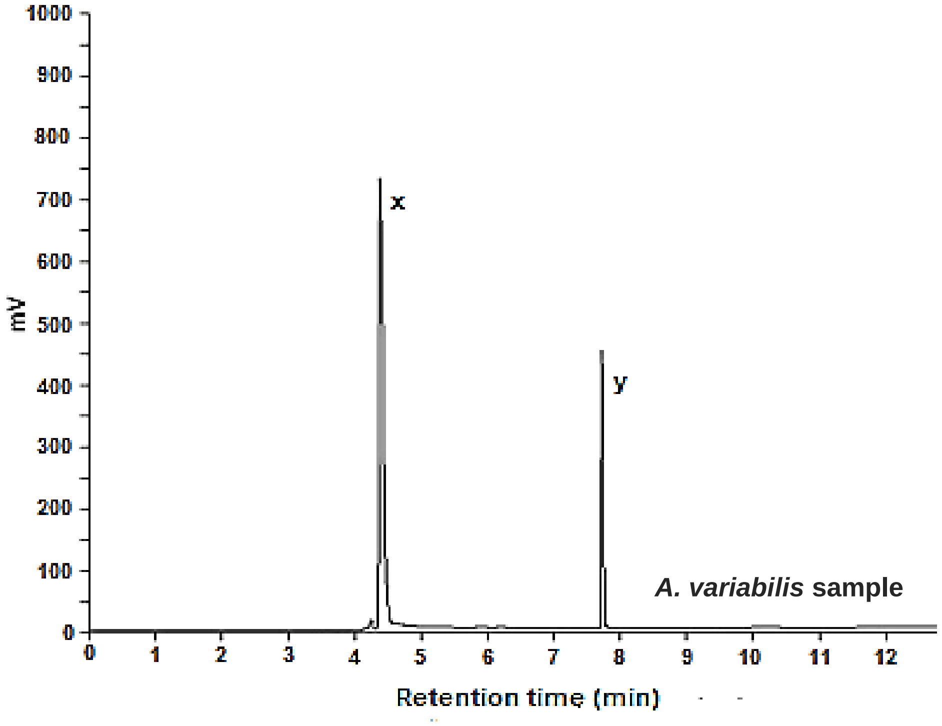

(b)

Supplementary Figure S4. GC chromatograms of **(a**) standard PHB and **(b)** *A. variabilis* sample. x: β-hydroxybutyric acid propyl ester, and y: benzoic acid propyl ester (internal standard).

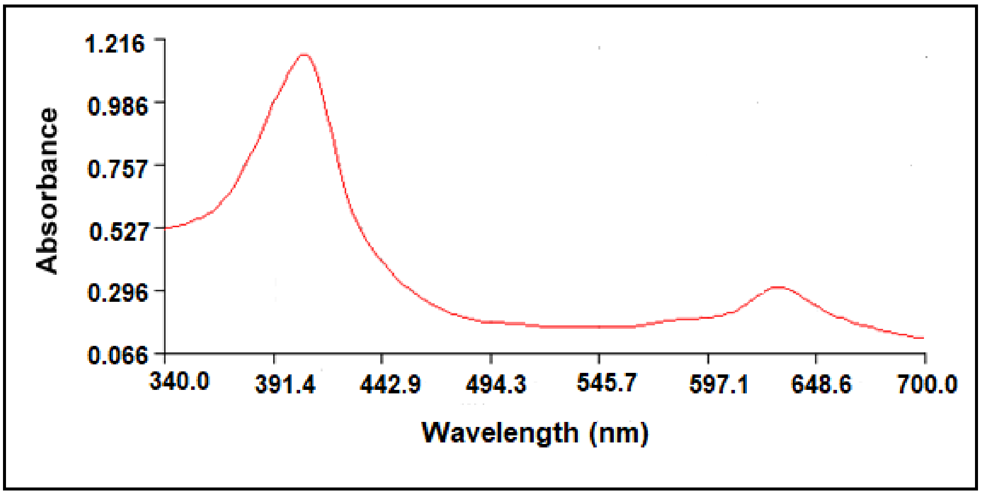

(a)

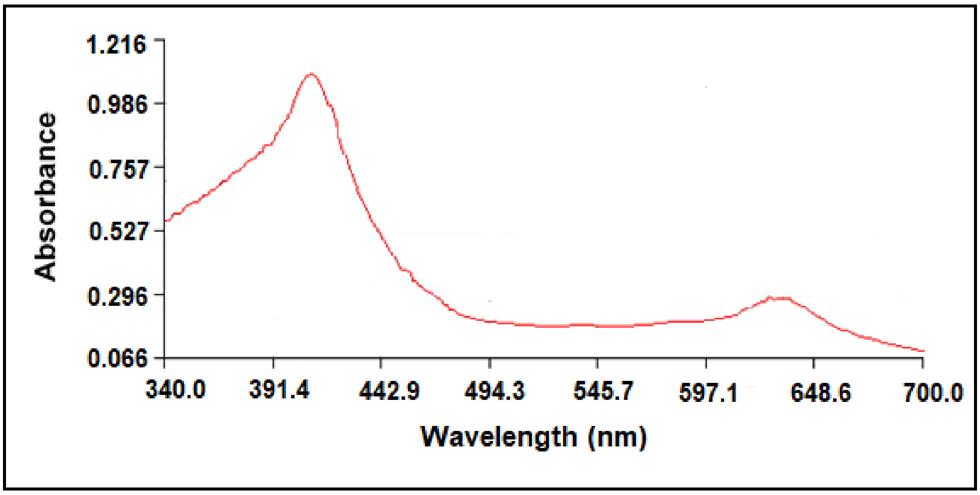

(b)

Supplementary Figure S5. Absorption spectra of **(a)** standard SCC and **(b)** prepared sample, obtained using UV-Vis spectrophotometer.

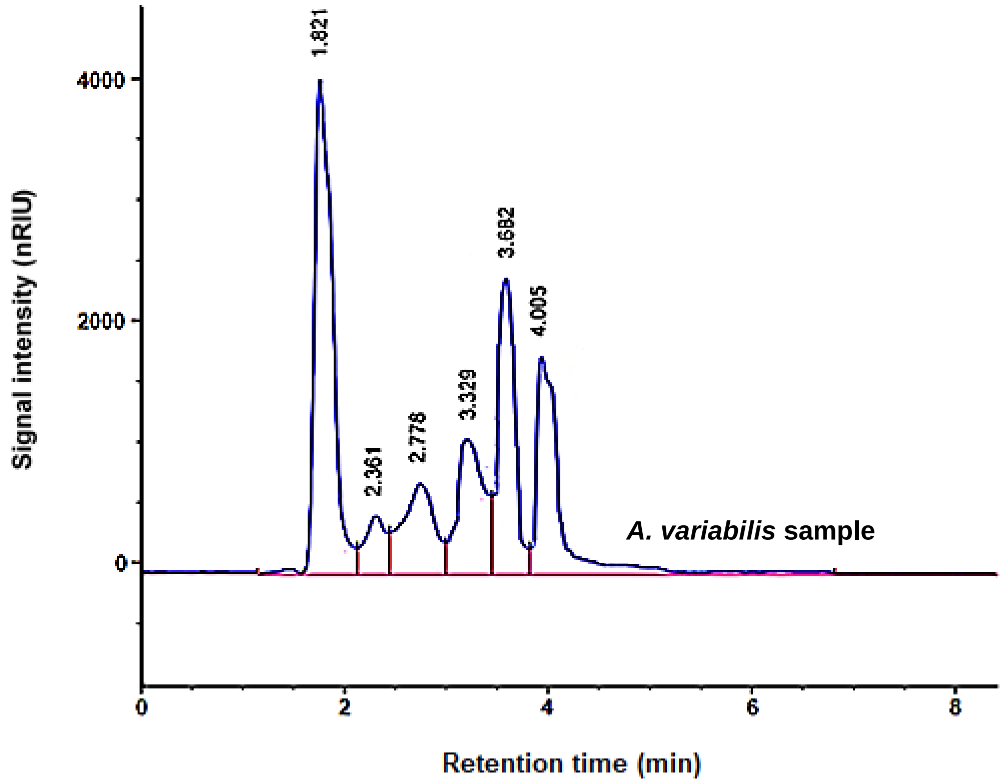

Supplementary Figure S6. Elution pattern of different sugar moieties in the hydrolyzed EPS from *A. variabilis*, obtained by HPLC analysis.

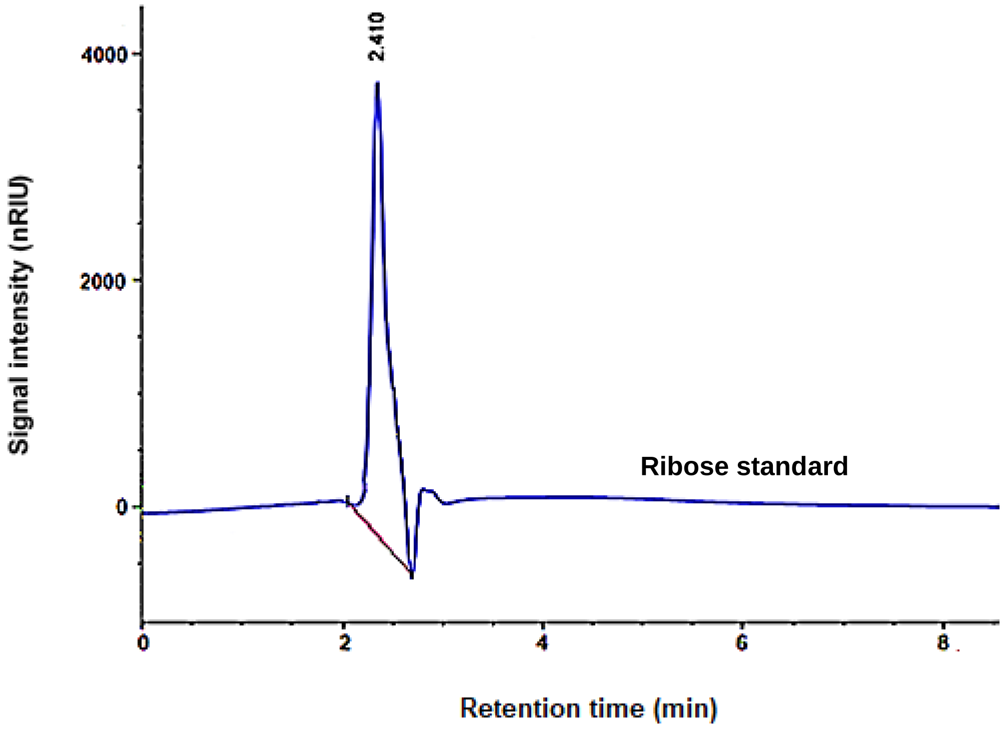
2.410
4000
2000
0
Signal intensity (nRIU)
Ribose standard
0
2
4
6
8
Retention time (min)

(a)

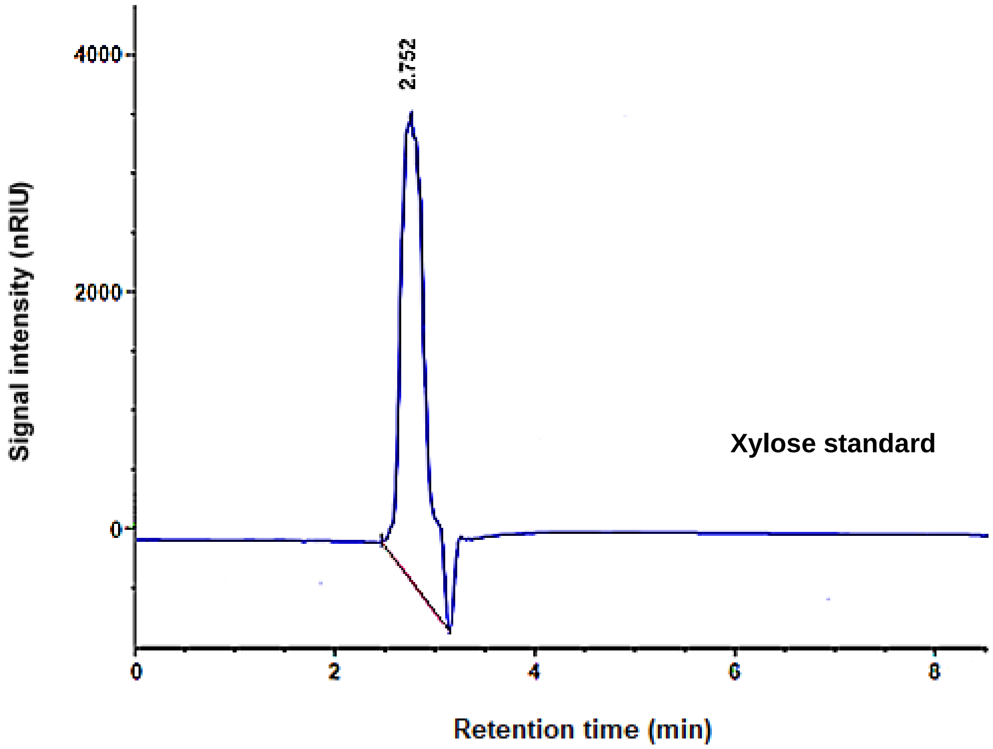
2.752
4000
2000
0
Signal intensity (nRIU)
Xylose standard
0
2
4
6
8
Retention time (min)

(b)

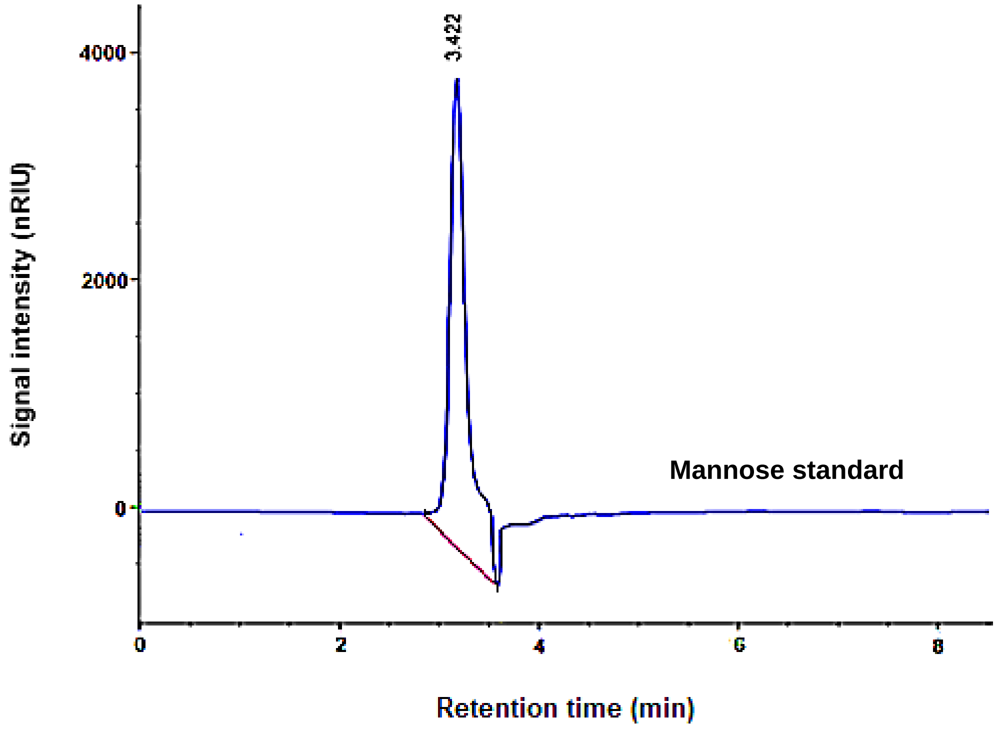
3.422
4000
2000
0
Signal intensity (nRIU)
Mannose standard
0
2
4
6
8
Retention time (min)

(c)

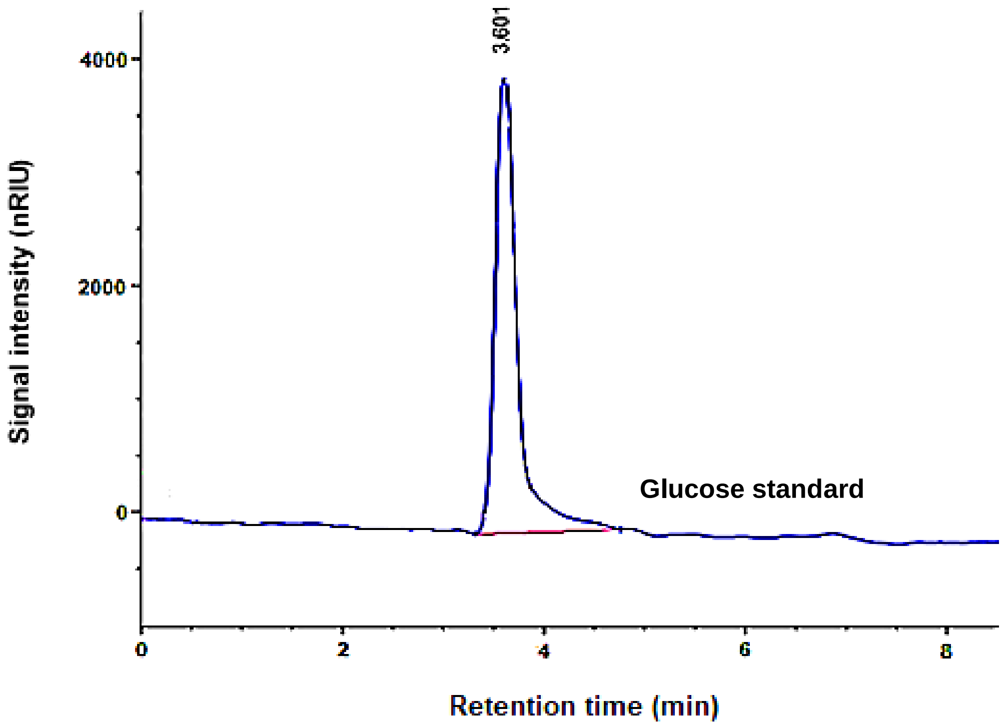
3.601
4000
2000
0
Signal intensity (nRIU)
Glucose standard
0
2
4
6
8
Retention time (min)

(d)

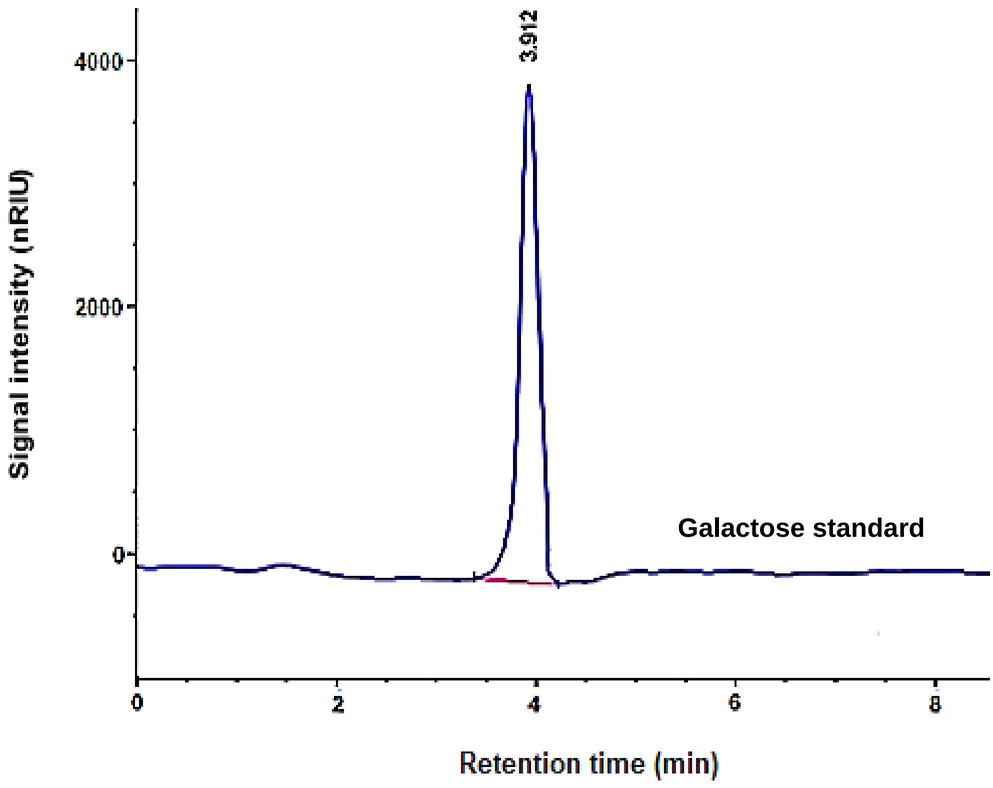

(e)

Supplementary Figure S7. Elution pattern of different sugar standards viz., **(a)** ribose, **(b)** xylose, **(c)** mannose, **(d)** glucose, and **(e)** galactose obtained by HPLC analysis.
